# Generation of labeled leaf point clouds for plants trait estimation

**DOI:** 10.1016/j.plaphe.2025.100071

**Published:** 2025-06-16

**Authors:** Gianmarco Roggiolani, Brian N. Bailey, Jens Behley, Cyrill Stachniss

**Affiliations:** a Center for Robotics, University of Bonn, Germany; b Department of Plant Science, University of Davis, United States; c Lamarr Institute for Machine Learning and Artificial Intelligence, Germany

**Keywords:** 3D plant phenotyping, Leaf trait estimation, Deep learning for agriculture

## Abstract

Today, leaf trait estimation remains a labor-intensive process. The effort to obtain ground truth measurements limits how accurately this task can be performed automatically. Traditionally, plant scientists manually measure the traits of harvested leaves and associate them with sensor data, which is key for training machine learning approaches and to automate the processes. In this paper, we propose a neural network-based method to generate synthetic 3D point clouds of leaves with their associated traits to support approaches for phenotyping. We use real-world leaf point clouds to learn how to generate realistic leaves from a leaf skeleton, which is automatically extracted. We use the generated leaves to fine-tune different leaf trait estimation methods. We evaluate our generated data using different trait estimation methods and compare the results to using real-world data or other synthetic datasets from agricultural simulation software. Experiments show that our approach generates leaf point clouds with high similarity to real-world leaves. Tuning trait estimation methods on our generated data improves their performance in the estimation of real-world leaves’ traits, making our data crucial for developing and testing data-driven trait estimation methods. Accurate trait estimation is key to understanding crop growth, productivity, and pest resistance, as leaf size directly influences photosynthesis, yield potential, and vulnerability to insects and fungal growth.

## 1 Introduction

The global demand for food, fuel, and fiber constantly increases due to the growing world population. Agricultural systems must meet this growing demand while producing resources more sustainably. The common practice of expanding farmland has reached its limit due to desertification, salinization, and soil erosion. The problem of increasing crop production is tackled by developing new crop varieties, with higher crop yield and toward resistance to stress and diseases [[1], [2], [3]]. Prior works [[4], [5], [6]] have linked crop productivity to different traits of the leaf morphology, such as the leaf width and length or its shape.

Phenotyping is the process of measuring traits of plants. Today, this task is still mainly performed by workers measuring observable traits manually, making it an expensive, time-consuming, and difficult-to-scale task [7]. This limitation is evident in agricultural datasets, which often provide only the average traits computed over a few manually selected leaves, reducing the granularity and accuracy of successive analyses. However, this is often not enough to evaluate approaches that aim to estimate per-plant traits and is even more problematic for training deep learning approaches, which usually require large amounts of labeled data for supervised training [8]. Obtaining such data is time-consuming, costly, and often a bottleneck for algorithm development.

In this paper, we tackle the problem of the lack of training data by developing a generative approach to produce leaf point clouds with a given length and width that we use to optimize approaches for the estimation of these leaf traits. Prior works [[9], [10], [11]] on trait estimation focused on leaf instance segmentation on images treating the number of leaves as the main trait. However, images provide a limited understanding of angles and curvatures, which are needed to estimate the length and width of bending leaves. 3D point clouds can better capture the geometry of the leaves, allowing for more accurate estimation of geometric leaf traits, such as the leaf width and length. Most of the approaches for 3D data are rule-based [[12], [13], [14], [15]] instead of data-driven [16,17] since the lack of data with reference traits does not allow for training of learning-based methods to estimate traits different from the number of leaves. However, such approaches still need fine-tuning to achieve satisfactory performance.

The main contribution of this paper is a novel approach for generating leaf point clouds with their associated leaf traits. Our work opens the road to the development, benchmarking, and comparison of next-generation trait estimation techniques previously limited by the lack of data. Unlike the traditional template leaf model – a mechanistic representation developed by expert plant scientists to capture the leaf morphology, we use a generative network trained on real-world data. As input, our network receives a point cloud representing a leaf skeleton with its traits as high-level descriptors. The network generates realistic point clouds of leaves as output. Training on real-world data allows us to generate leaves with distributions similar to real ones, without the need for additional expert knowledge for each different plant species. We generate new leaves by providing a skeleton of the desired length and width. In this article, we decompose the problem of trait estimation into two parts: firstly, a generative method produces leaf point clouds with their respective leaf width and length, and secondly, we use the generated data to optimize the parameters of a trait estimation approach. Further details on the problem decomposition can be found in the supplementary material. We compare our approach against other geometric and learning-based leaf generation methods, showing that our generated leaf point clouds are similar to the real-world leaf distribution. Then, since our approach tackles the problem of the lack of data for trait estimation methods, we show that tuning different off-the-shelf trait estimation approaches on our generated data significantly improves the accuracy and precision of real-world leaf trait estimation. We evaluate our approach on multiple datasets of different crop species. In sum, we make two key claims: (i) using our generated leaves to tune trait estimation approaches performs better than using other generated or real-world leaf point clouds; and (ii) all generated leaf point clouds respect the leaf traits we condition on and have a high probability of being sampled from the real-world leaf distribution. We plan to make our code publicly available to enable further development of trait estimation methods.

## 2 Related work

The problem of trait estimation in agriculture is still largely tackled by manual measurements performed by domain experts. This process is expensive, labor-intensive, and prone to introduce biases in the collected data. For example, bigger leaves are easier to identify and remove, leading to them being over-represented and resulting in a biased estimate of the trait distributions. As with many other agricultural tasks, trait estimation can be automatized using robotic platforms equipped with perception systems [[18], [19], [20]]. These systems capture the leaf data and analyze it without human intervention at a fine-grained scale and in a non-destructive fashion, i.e., without the need to remove the leaf from the plant.

**2D Trait Estimation:** Lately, several computer vision approaches have been developed to estimate phenotypic and functional traits from 2D images. Most of them focused on leaf segmentation and counting, initially using heuristic approaches and later based on neural networks. Multiple geometric segmentation techniques have been employed to segment single leaves, such as the region growing algorithm in the work by Pape et al. [21], adaptive thresholding in the work by Bai et al. [22], and the Sobel operator used by Wang et al. [23]. Heuristic approaches often rely on tuning several hyperparameters to obtain satisfactory performance. Deep learning methods typically require fewer manually tuned parameters, as their weights are optimized during training with labeled data. Deep learning approaches using convolutional neural networks have been shown to outperform heuristic methods for leaf segmentation, especially when they exploit knowledge about the plant structure [10,24]. However, these approaches only count the number of leaves or estimate the leaf area [25]. Because of the projective view of the images, it is challenging for any image-based approach to estimate per-leaf traits, such as the leaf length and width, which provide more information about crop growth and pest resistance.

**3D Trait Estimation:** Using 3D data, i.e., point clouds or meshes, allows for estimating more complex leaf traits, such as widths and lengths of the leaves, which are hard to determine using images only. The potential of 3D data for plant trait estimation has already been shown by several approaches developed in the context of horticulture [26,27], where automatic estimation of the size and color of single fruits is crucial for automatic harvesting and yield estimation. In the context of leaf trait estimation, many works still focus on controlled environments [12,13], assuming perfect 3D data, with only one plant in the scene, and thus, with little to no occlusion. The problem of handling data that is not fully visible because of occlusions or missing viewpoints severely impacts the ability to segment the leaves and estimate their widths and lengths. This limits the application of such methods in complex real-world scenes.

Marks et al. [15] tackle this limitation by means of template reconstructions. They propose using a deformable template mesh to reconstruct the leaves, even in the case of missing parts due to self-occlusions or occlusions from other plants. Once the leaf is reconstructed in 3D without missing parts, they estimate the position of the leaf center, tip, and right and left corners. The majority of the approaches use the geometric structure of the data and the knowledge about the appearance of the plants to segment the leaves and estimate the relevant traits. The lack of ground truth traits per single leaf limits the deployment of deep-learning approaches. Recent generative approaches could solve this problem by producing synthetic annotated data for supervised learning.

**Generated Data for the Agricultural Domain:** In recent years, several works have proposed to use artificially generated data in the agricultural domain. Most of these approaches generate images to train networks for crop-weed segmentation [28,29]. In the context of 3D data generation, Helmrich et al. [30] propose a pipeline requiring user-defined parameters for the plant, the root system, and the functional model of the plants. This makes the approach suitable for different functional and structural analyses but requires expert knowledge for modeling the plant and setting parameters. Additionally, they generate the 3D agricultural scene only to export images of the scene. The work by Bailey [31] generates synthetic fields of different crop species. Their simulation software focuses on functional traits, i.e., radiation, photosynthesis, and water conduction. Similarly, expert knowledge is required to build the plant and set the parameters for each crop species. None of the works can exploit real-world data collected automatically, for example, by a robot or mobile sensing system. Instead, they require access to manually measured traits to set the parameters.

Our work falls into the category of methods that generate data for the agricultural domain. We propose an approach to automatically generate point clouds of leaves with known widths and lengths. In line with this methodological approach, Choi et al. [32] use the 3D Plant simulator Helios [31] to simulate a 3D agricultural scene from which they create a synthetic image dataset for training networks. As Helmrich et al. [30], they build 3D scenes only to export images of the scene as a dataset for training deep-learning methods. Our main contribution is the generation of leaf point clouds annotated for leaf trait estimation without the requirement for labeled data or expertise that can be learned from real-world data in an unsupervised fashion. We also show how directly using our generated point clouds enables more accurate leaf trait estimation for real-world leaves.

## 3 Problem formulation

We formally define the problem before explaining our proposed method for generating point clouds of leaves. Leaf trait estimation is performed by a method that we express as a function

(1) $$f\left(P_{i},\theta \right)={\hat{t}}_{i},$$

where $P_{i}$ is the input leaf point cloud, ***θ*** are the approach's parameters, ${\hat{t}}_{i}\in R^{T}$ is the vector of *T* estimated traits for the input leaf $P_{i}$, each one a scalar. We can find the optimal parameters of the approach given a dataset of *D* leaf point clouds with traits $D={\left\{\left(P_{i},t_{i}\right)\right\}}_{i=1}^{D}$ as

(2) $$\theta_{D}^{*}=\arg\;\underset{\theta \in \Theta }{\min}\;\underset{\left(P_{i},t_{i}\right)\in D}{\sum }e\left(f\left(P_{i},\theta \right),\;t_{i}\right)=\arg\;\underset{\theta \in \Theta }{\min}\;\underset{t_{i}\in D}{\sum }e\left({\hat{t}}_{i},\;t_{i}\right),$$

where Θ is the set of possible parameters ***θ***, and $e\left({\hat{t}}_{i},\;t_{i}\right)$ is a function computing the error between the estimated traits ${\hat{t}}_{i}$ and the ground truth traits **t**_*i*_ in D. The error function *e* used may depend on the estimated traits, e.g., the cosine similarity is appropriate for the angle between the leaf and the plant stem but not for the length of the leaf blade. As for any optimization procedure, the final performance of the trait estimation approach *f* depends on the dataset's completeness and reliability. As already mentioned in Sec. 1, the real-world agricultural datasets $D_{\text{real}}$ associates multiple leaf point clouds $P_{i}^{\text{real}}$ with the same average traits computed over a few manually selected leaves. We want to tackle the problem of *generating a dataset* with known traits *for each* leaf point cloud. We introduce the generative problem as defining a function

(3) $$g\left(t_{i}\right)={\hat{P}}_{i},$$

that generates leaf point cloud ${\hat{P}}_{i}$ for given traits **t**_*i*_. In this way, we can generate a new dataset

(4) $$D_{g}={\left\{\left(g\left(t_{i}\right),t_{i}\right)\right\}}_{i=1}^{D}.$$

This new dataset $D_{g}$, with per-leaf ground truth traits **t**_*i*_ is used to find the best parameters $\theta_{D_{g}}^{*}$ for any given trait estimation method *f*. The generative function *g*(**t**) must generate realistic data to obtain a valuable dataset and, thus, parameters $\theta_{D_{g}}^{*}$ that perform well on real-world point clouds.

## 4 Materials and methods

**Fig. 1:**
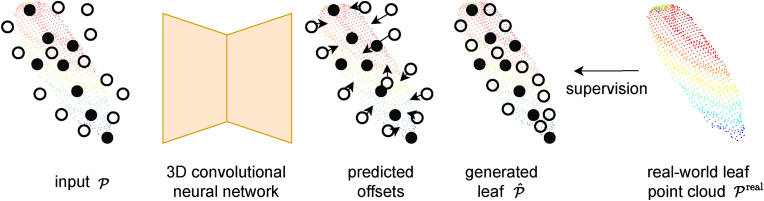
Overview of our approach. We show the input point cloud P made up from the skeleton points in and in white the points sampled from the GMM. Our network predicts per-point offsets depicted as arrows. Adding the offsets to the points' positions we obtain P. We supervise the network using real leaf point clouds $P^{\text{real}}$.

We propose a novel approach that generates synthetic leaf point clouds $\hat{P}$ with known traits. Instead of relying on a mechanistic model, we train a 3D convolutional neural network to generate synthetic leaf point clouds of desired traits **t**. This is the *g* function of our problem formulation in Eq. (3). An overview of our approach is shown for an exemplary tomato leaf in Fig. 1.

In our work, we consider the leaf blade length and width as traits. Such traits are intrinsic in the leaf skeleton point cloud extracted from real-world leaves. We do not need their actual values to train our network. In Sec. 4 .1, we illustrate how to obtain the skeleton point clouds S from the point clouds of real leaves $P^{\text{real}}$. We do not pose constraints on how to acquire the real-world point clouds. The datasets we use have either been created by means of a laser scanning system with sub-millimeter accuracy or using photogrammetric reconstruction including bundle adjustment [33] on a set of images of the field. Then, in Sec. 4 .2, we describe how we add more points to the skeleton point cloud S to capture the shape of the whole leaf and obtain the input P for our network. We then explain the network's architecture. In Sec. 4 .3, we explain the loss we minimize during our training. Sec. 4 .4 describes how we build skeletons and compute accurate traits since we know the functions and limits that define the skeleton. Our network uses these skeletons to generate new leaf point clouds of known leaf blade length and width.

### 4.1 Extraction of leaves skeletons

The first step of our approach extracts skeleton point clouds S of real-world leaves $P^{\text{real}}$. We use two existing approaches by Marks et al. [15] and by Magistri et al. [34] to show that our approach can work with different skeleton extraction methods. The skeleton serves as a structural backbone of the leaf, capturing the petiole, the main axis along the leaf length, and the lateral axis along the leaf width. In the literature, there is no universal definition of leaf width. For the approach by Marks et al. they define it on their template, while for the approach by Magistri et al., we define it as the width at the midsection of the leaf.

**Fig. 2:**
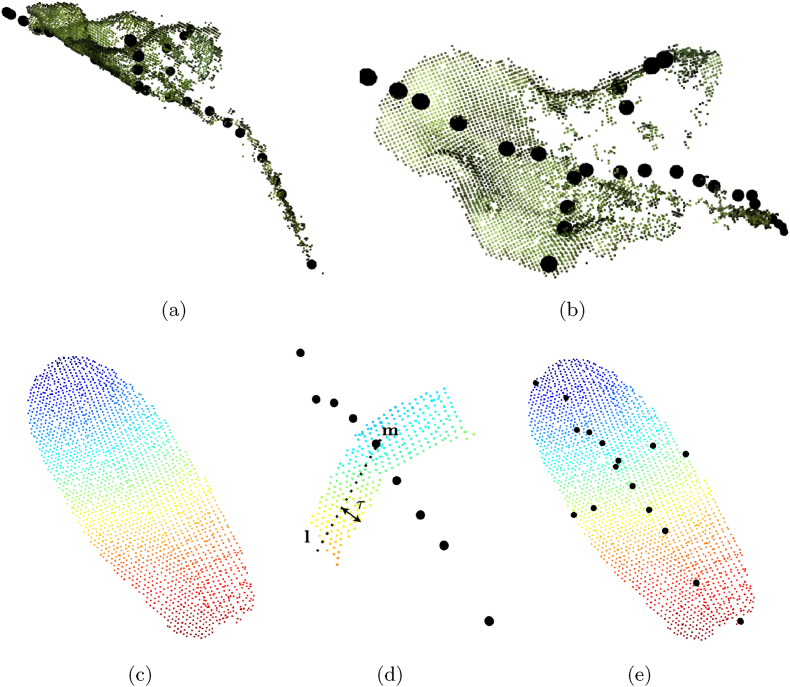
We show the extracted skeleton using the approach by Marks et al. [15] (top) for a sugar beet leaf, and the approach by Magistri et al. [34] (bottom) with our adaptation for a maize leaf. The skeleton S is always shown in black circles. We show the view from the side (a) and the top (b). For the maize leaf in (c), we show the skeleton extracted along the main axis and the points of the leaf slice cut around **m** in (d). In (e), the final skeleton with main and lateral axes.

Marks et al. [15] manually define all the points and faces for a template mesh of a leaf that they deform to fit it to real leaf point clouds $P^{\text{real}}$. They also define which points in the template represent the center, tip, right and left corners of the leaf, and which subsets of points represent the main axis, the lateral axis, and the petiole. The template targets sugar beet plants and new template meshes are needed for each new crop species. Since they define the points in the template belonging to the main and lateral axis, after fitting the template mesh to the leaf point cloud $P^{\text{real}}$, we use the positions of such points as points for our skeleton point cloud S. The top row of Fig. 2 shows the extracted skeleton for one exemplary sugar beet leaf.

We use the approach by Magistri et al. [34] to extract the skeleton point clouds S of leaves of tomato and maize plants. The main limitation of their approach is that it only provides the points of the skeleton along the main axis of the leaf, which usually represents the leaf length. Thus, their approach does not detect the points of the lateral axis, i.e., along the width direction of the leaf. Magistri et al. [34] generate a chain of *n* 3D points and fit it to the leaf point cloud. In the bottom row of Fig. 2, we show how to use their approach to also compute the points of the skeleton along the width direction of the leaf. After computing the *n* points of S along the main axis, we compute the median point $m\in R^{3}$, and the direction **n** of the lateral axis of the leaf as the second principal component extracted using principal component analysis on $P^{\text{real}}$. We then cut a slice of the leaf around **m**, preserving all points in the direction of **n** and removing points whose distance from the line **l** ​= ​**m** ​+ ​*c***n**, where c∈R, is larger than *τ*. This slice represents the central section of the leaf, from which we want to extract the points representing its width. In Fig. 2 (d), we show the skeleton along the main axis over the points that we keep at the end of this step. We then apply the approach only on the points in the area of interest to detect the skeleton points in the direction of the leaf width. The final result, which we obtain by combining the points from this two-step approach, is shown in Fig. 2 (e).

### 4.2 From skeletons to network outputs

**Fig. 3:**
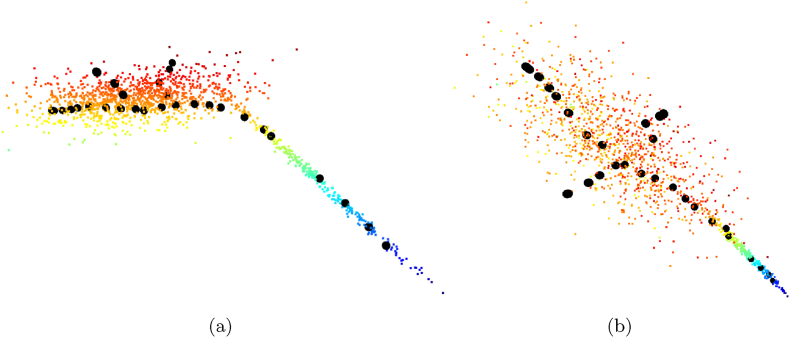
We show the point cloud P, i.e., the input of our generative function *g*. The skeleton points are shown in black circles. The other points are sampled from the Gaussian Mixture Model fitted on the skeleton. We show the view from the side (a) and the top (b).

We generate the leaves using a neural network, specifically a 3D U-Net [35] based on KPConv [36]. At the end of the previous section, we obtained the skeleton point cloud S with $\tilde{N}$ points $P_{\text{skeleton}}\in R^{\tilde{N}\times 3}$ representing the leaf skeleton. To reconstruct a complete leaf Y, we add extra points beyond those of the skeleton to have enough points to ensure a realistic shape. We call *N* the total number of points in the point cloud P that we use as input for the network. We set $N=\tilde{N}+\delta \tilde{N}$, where $\delta \in Z^{+}$ is a parameter that scales the number of total points according to the number of points in the skeleton S. We sample the extra points $P_{\text{sampled}}\in R^{\delta \tilde{N}\times 3}$ from a Gaussian mixture model (GMM) [37] fitted to the original skeleton points **P**_skeleton_. A GMM is a probability distribution of density

(5) $$p\left(p_{\text{sampled},u}\right)=\overset{J}{\underset{j=1}{\sum }}\pi_{j}\;N\left(p_{\text{sampled},u};\mu_{j},\Sigma_{j}\right),$$

where $p_{\text{sampled},u}\in R^{3}$ is the position of the *u* ​− ​th sampled 3D point, *J* is the number of distributions in the mixture, *π*_*j*_ is the probability of selecting the *j* ​− ​th distribution, $\mu_{j}\in R^{3}$ is the mean and $\Sigma_{j}\in R^{3\times 3}$ is the covariance of the *j* ​− ​th distribution. When collecting the real point clouds $P^{\text{real}}$ we must know if they also include the petiole, when the petiole is present we set *J* ​= ​2, otherwise we set *J* ​= ​1. We need two modes when the petiole is present because we expect one Gaussian to capture the petiole and one to capture the leaf surface. We now call P the point cloud obtained by adding the points **P**_sampled_ to those present in the skeleton point cloud S. We show the resulting point cloud P in Fig. 3 for a sugar beet leaf, where one Gaussian is fitted to the petiole and one to the leaf blade.

The output of our 3D U-Net is an offset vector $o\in R^{3}$ for each point in the input point cloud P. We compute the positions of each *u* ​− ​th point ${\hat{p}}_{u}$ in the output point cloud $\hat{P}$ as ${\hat{p}}_{u}=p_{u}+o_{u}$.

### 4.3 Loss functions

The objective of our generative function *g* in Eq. (3), is to generate leaf point clouds $\hat{P}$ respecting the traits **t** defined by the skeletons S, and whose points distribution is close to the real-world one. To achieve this, we combine different loss functions in the training procedure. We divide the loss functions into two main groups. The first group consists of reconstruction loss functions defined on the real-world point cloud $P_{i}^{\text{real}}$ from which we extract the skeleton $S_{i}$ and the output point cloud ${\hat{P}}_{i}$. The second group consists of loss functions based on the points distribution for all $P_{i}^{\text{real}}\in D_{\text{real}}$. The first group of loss functions aims to produce a leaf point cloud $\hat{P}$, which respects its skeleton S, looks like the original point cloud $P^{\text{real}}$ from which S was extracted, and has a smooth surface. The second group of loss functions forces the output point cloud $\hat{P}$ to have a similar point distribution with respect to the point distribution in the real leaf point clouds $P^{\text{real}}$. Our approach minimizes the total loss

(6) $$L=\lambda_{1}L_{\text{skeleton}}+\lambda_{2}L_{\text{chamfer}}+\lambda_{3}L_{\text{edges}}+\lambda_{4}L_{\text{smooth}}+\lambda_{5}\left(L_{\text{CMMD}}+L_{\text{FID}}+L_{\text{PR}}\right),$$

where we weight the different loss functions using $\lambda_{a},a\in \left\{1,2,3,4,5\right\}$. The reconstruction loss functions are $L_{\text{skeleton}}$, $L_{\text{chamfer}}$, $L_{\text{edges}}$, and $L_{\text{smooth}}$, while $L_{\text{CMMD}}$, $L_{\text{FID}}$, and $L_{\text{PR}}$ are distribution loss functions.

**Reconstruction Loss Functions.** The reconstruction loss functions use the generated leaf point cloud $\hat{P}$ and the real leaf point cloud $P^{\text{real}}$ from which we extracted the skeleton S. Their main purpose is to let the network learn how to generate a leaf respecting the traits **t** defined by S. The first term $L_{\text{skeleton}}$ forces the network to keep the skeleton points **P**_skeleton_ in their original positions, thus preserving the desired traits **t**. To keep the skeleton points fixed, we enforce that the offsets predicted for those points have all components equal to zero, resulting in the loss term

(7) $$L_{\text{skeleton}}=\overset{\tilde{N}}{\underset{i}{\sum }}|o_{i}|1\left[p_{i}\in S\right],$$

where $1\left[p_{i}\in S\right]$ is an indicator function evaluating to 1 when the points **p**_*i*_ belongs to S.

The second term $L_{\text{chamfer}}$ is the Chamfer distance. In the literature, this distance is used to evaluate the distance between two sets of points [26,38]. We include it in our loss to enforce that the points of the generated point cloud $\hat{P}$ are as close as possible to the points of the real-world point cloud $P^{\text{real}}$:

(8) $$L_{\text{chamfer}}=\underset{p^{\text{real}}\in P^{\text{real}}}{\sum }\underset{\hat{p}\in \hat{P}}{\min}{\left|\left|p^{\text{real}}-\hat{p}\right|\right|}_{2}+\underset{\hat{p}\in \hat{P}}{\sum }\underset{\hat{p}\in \hat{P}}{\min}{\left|\left|p^{\text{real}}-\hat{p}\right|\right|}_{2},$$

where $p^{\text{real}}\in R^{3}$ is a point belonging to the real-world leaf point cloud $P^{\text{real}}$. We compute the Chamfer loss in both directions, i.e., we compute the closest point in $\hat{P}$ for each **p**^real^ and the closest point in $P^{\text{real}}$ for each $\hat{p}$.

The third term $L_{\text{edges}}$ is a regularization loss to enforce that the distance between neighboring points in $\hat{P}$ is close to a user-defined value $\bar{l}$. This loss enforces that points are evenly distributed in space, penalizing areas that are too sparse or too dense. We compute a *k*-NN graph over the output point cloud $\hat{P}$ defining a maximum distance *d*_max_ for two points to be connected, i.e., we define an edge $e_{u,v}\in E$ of length $l_{u,v}={\left|\left|{\hat{p}}_{u}-{\hat{p}}_{v}\right|\right|}_{2}$ between points ${\hat{p}}_{u}$ and ${\hat{p}}_{v}$ if ${\hat{p}}_{v}\in {NN}_{k}\left({\hat{p}}_{u}\right)$, where ${NN}_{k}\left({\hat{p}}_{u}\right)$ is the set of *k* neighbors with distance from ${\hat{p}}_{u}$ smaller than *d*_max_. We then compute the loss as

(9) $$L_{\text{edges}}=\frac{1}{N\;\sum_{u}|{NN}_{k}\left({\hat{p}}_{u}\right)|}\overset{N}{\underset{u=1}{\sum }}\underset{v\in {NN}_{k}\left({\hat{p}}_{u}\right)}{\sum }\left|l_{u,v}-\bar{l}\right|,$$

where $\left|{NN}_{k}\left({\hat{p}}_{u}\right)\right|$ is the cardinality of the nearest neighbors of point ${\hat{p}}_{u}$, and $\left|l_{u,v}-\bar{l}\right|$ is the absolute distance of edge *l*_*u*,*v*_ from $\bar{l}$.

The last term $L_{\text{smooth}}$ is a regularization loss to enforce the generated leaf point cloud $\hat{P}$ to have a smooth surface. This loss acts like a denoising operation and penalizes single points that are too far from their neighbors and that would lead to sharp changes of the leaf surface. We use the edges computed before via the *k*-NN graph to compute the Laplacian matrix $L\in R^{N\times N}$ as

(10) $$L_{u,v}=\left\{\begin{array}{ll} -1\; & \text{if}\;u=v\\ \frac{1}{\left|NN_{k}\left({\hat{p}}_{u}\right)\right|}\; & \text{if}\;\exists e_{u,v}\in \;E\\ 0\; & \text{otherwise} \end{array}\right)$$

Then, we compute the Laplacian smoothing objective as $Q=L\hat{P}$, where $\hat{P}\in R^{N\times 3}$ is a matrix where each row is a point ${\hat{p}}_{u}\in \hat{P}$. We define the loss as

(11) $$L_{\text{smooth}}=\overset{N}{\underset{u=1}{\sum }}\left|Q_{u}\right|,$$

where $Q_{u}\in R^{3}$ is the *u* ​− ​th row of **Q**.

**Distribution Loss Functions.** The second group of loss functions enforces that the distribution of the points in our generated dataset $D_{\text{ours}}$ of size *D*_ours_, is as close as possible to the points distribution of the real-world dataset $D_{\text{real}}$ of size *D*_real_. We use three commonly used metrics for data generation and phrase them as losses. The first term $L_{\text{CMMD}}$ is the maximum mean discrepancy of the 3D CLIP embeddings [39] given by

(12) $$\begin{array}{ll} L_{\text{CMMD}} & =\frac{1}{D_{\text{ours}}\left(D_{\text{ours}}-1\right)}\overset{D_{\text{ours}}}{\underset{i=1}{\sum }}\overset{D_{\text{ours}}}{\underset{j\neq i}{\sum }}\left\langle v_{r,i}^{\text{CLIP}},v_{r,j}^{\text{CLIP}}\right\rangle \\ & +\frac{1}{D_{\text{real}}\left(D_{\text{real}}-1\right)}\overset{D_{\text{real}}}{\underset{i=1}{\sum }}\overset{D_{\text{real}}}{\underset{j\neq i}{\sum }}\left\langle v_{f,i}^{\text{CLIP}},v_{f,j}^{\text{CLIP}}\right\rangle \\ & -\frac{2}{D_{\text{ours}}D_{\text{real}}}\overset{D_{\text{ours}}}{\underset{i=1}{\sum }}\overset{D_{\text{real}}}{\underset{j=i}{\sum }}\left\langle v_{r,i}^{\text{CLIP}},v_{f,j}^{\text{CLIP}}\right\rangle , \end{array}$$

where $v_{r,i}^{\text{CLIP}}$ and $v_{f,j}^{\text{CLIP}}$ are the CLIP embeddings of the *i* ​− ​th real-world point cloud and of the *j* ​− ​th generated point cloud. Jayasumana et al. [40] were the first to propose the use of CLIP embeddings, initially for the evaluation of generated images. Exploiting the work by Hegde et al. [39] who provide 3D CLIP embeddings trained on point cloud-image-caption triplets, we compute the CMMD on point clouds.

The second term $L_{\text{FID}}$ comes from the Fréchet inception distance (FID). As for the previous term, we first compute embeddings for all point clouds, both the real-world $D_{\text{real}}$ and the generated ones $D_{\text{ours}}$. We can use the model by Hegde et al. [39] to obtain CLIP embeddings or any other neural network to extract embedding from the point clouds. Once we have the embeddings **v**_*r*,*i*_ for all the real-world point clouds and **v**_*f*,*j*_ for all the generated point clouds, we fit Gaussians $N\left(\mu_{r},\sigma_{r}\right)$ and $N\left(\mu_{f},\sigma_{f}\right)$ to the two embedding distribution. We compute the FID as

(13) $$L_{\text{FID}}=\|\mu_{r}-\mu_{f}{\|}_{2}+\text{tr}\left(\Sigma_{r}+\Sigma_{f}\;-\;2\sqrt{\Sigma_{r}\Sigma_{f}}\right),$$

where **Σ**_*r*_ and **Σ**_*f*_ are the covariance matrices of two distributions, and tr$\left(\cdot \right)$ is the trace operation over the matrix.

The third term $L_{\text{PR}}$ comes from the precision and recall metrics. These metrics have been extended for evaluating generative approaches by Kynkäänniemi et al. [41]. We shortly explain how the metrics are computed and how we adapt them to use them as losses. As for the previous distribution loss functions, we need point cloud embeddings. We call Φ_*r*_ and Φ_*f*_ the sets of features extracted from the real and generated point clouds. For each set, we estimate a manifold in the feature space sampling a set of points and surrounding each with a hypersphere that reaches its *k* ​− ​th nearest neighbor. We then evaluate whether an embedding **v** is inside the volume estimated from the set of features Φ as

(14) $$b\left(v,\Phi \right)=\left\{\begin{array}{l} 1,\;\text{if}\;\;\exists_{v^{\prime }\in \Phi }:\|v-v^{\prime }{\|}_{2}\;<\|v^{\prime }-{NN}_{k}\left(v^{\prime },\Phi \right){\|}_{2}\;\\ 0,\;\text{otherwise}\; \end{array}\right)$$

where NN_*k*_(**v**′, Φ) returns the *k*-th nearest embedding of **v**′ from Φ. We now compute the precision Pr and recall R as

(15) $$\text{Pr}=\frac{1}{\left|\Phi_{f}\right|}\underset{v_{f}\in \Phi_{f}}{\sum }b\left(v_{f},\Phi_{r}\right)$$

(16) $$\text{R}=\frac{1}{\left|\Phi_{r}\right|}\underset{v_{r}\in \Phi_{r}}{\sum }b\left(v_{r},\Phi_{f}\right).$$

In contrast to the previous loss functions, which are distances, we cannot use the precision and recall as they are, since we aim to maximize them. Thus, we define the precision-recall loss as

(17) $$L_{\text{PR}}=\log_{10}\left(\frac{1}{\text{Pr}+\epsilon }\right)+\log_{10}\left(\frac{1}{\text{R}+\epsilon }\right),$$

where *ϵ* is a small value to ensure numerical stability. In the original paper [41] the authors noticed that the score is inaccurate when measuring the quality of a generated sample that falls into an area of the manifold where only a few real samples are present. Thus, they introduce the realism score

(18) $$\text{Realism}\left(v_{g},\Phi_{r}\right)=\underset{v_{r}}{\max}\left\{\frac{{\left|\left|v_{r}-{NN}_{k}\left(v_{r},\Phi_{r}\right)\right|\right|}_{2}}{{\left|\left|v_{g}-v_{r}\right|\right|}_{2}}\right\},$$

which is used to filter out elements in such sparse areas of the manifold. The higher the minimum realism score the more we are pruning our manifold Φ_*r*_, thus yielding accurate and higher scores.

Since the real-world data distribution does not change during training, we can easily pre-compute the target values for all the distribution loss functions $L_{\text{CMMD}},\;L_{\text{FID}},\;\text{and}\;L_{\text{PR}}$, i.e., **v**_*r*_, $N\left(\mu_{r},\sigma_{r}\right)$, and Φ_*r*_.

### 4.4 Generating new leaves

Our generative model *g* takes as input the desired leaf length and width. However, our network needs as input a point cloud P computed from a skeleton point cloud S, as explained in Sec. 4.2. Thus, we need to define how to build a skeleton point cloud S without extracting it from a real-world leaf. As mentioned in Sec. 4 .1, the skeleton consists of three parts: petiole, main axis, and lateral axis. We construct the skeleton in the 3D Cartesian frame, building the main axis along the *x* direction and the lateral axis along the *y* direction. The petiole is a line

(19) $$z\left(x\right)=\alpha \;x,\;x\in \left[x_{\text{min}},0\right],$$

where $\alpha ∼U\left(\frac{\pi }{6},\frac{\pi }{3}\right)$ and $x_{\text{min}}∼U\left(-1,-0.25\right)$. Since we want a 3D point cloud, all these points still need a *y* coordinate, which we fix to 0. The petiole can be removed from the generative procedure when it's known that the petiole is not present in the training data $D_{\text{real}}$. We do not use the petiole for the maize leaves since the petiole is not present in the used real-world point clouds $P^{\text{real}}$. The central axis is defined as a hyperbolic tangent

(20) $$z\left(x\right)=\frac{e^{x}-e^{-x}}{e^{x}+e^{-x}},\;x\in \left[0,1\right],$$

where we clamp the hyperbolic tangent between *x* ​= ​0, where the petiole starts, and *x* ​= ​1. All points have *y* ​= ​0. We then scale the axis to different sizes.

We define the point where the central axis intersects the lateral axis as $p_{\text{cross}}={\left[x_{\text{cross}},0,z_{\text{cross}}\right]}^{T}$, where $x_{\text{cross}}∼U\left(0.25,0.75\right)$ and *z*_cross_ is given by Eq. (20). We use a parabolic function,

(21) $$z\left(y\right)=a\;y^{2}+b\;y+c$$

to represent the lateral axis, where all points have *x* ​= ​*x*_cross_. To compute the parabola coefficients *a*, *b*, and *c*, we need 3 points. One point is **p**_cross_, and two are the extremes on the right and left. We define them as

(22) $$\begin{array}{ll} p_{r} & ={\left[x_{\text{cross}},0.5,z_{\text{cross}}+z_{\text{r}}\right]}^{⊤}\\ p_{l} & ={\left[x_{\text{cross}},-0.5,z_{\text{cross}}+z_{\text{l}}\right]}^{⊤}, \end{array}$$

where *z*_r_ and *z*_l_ are two distinct values sampled from U(−0.25,0.25). It is important to note that the width of the leaf projected on the *y* axis is 1 and we can scale it to different sizes. The final skeleton is the collection of points sampled along the curves in Eq. (19), Eq. (20), and Eq. (21). We then scale this parametric skeleton by multiplying all *x* coordinates of the points for the length scaling factor *s*_*l*_, and all the *y* coordinates for the width scaling factor *s*_*w*_. The scaling factors are the only user-defined parameters that influence the length and width of the generated leaves. Thanks to the different randomly sampled parameters *α*, *x*_min_, *x*_cross_, *z*_r_, and *z*_l_, we obtain a large variety of leaves whose lengths and widths are centered on the user desired dimensions.

We compute the final length and width of the leaf using the formula for the computation of arc lengths. The width of leaf *L*_width_ is computed as

(23) $$L_{\text{width}}=\int_{-s_{w}}^{s_{w}}\sqrt{z^{\prime }{\left(y\right)}^{2}+x^{\prime }{\left(y\right)}^{2}}\;dy=\int_{-s_{w}}^{s_{w}}z^{\prime }\left(y\right)\;dy=\int_{-s_{w}}^{s_{w}}\left(2ay+b\right)\;dythinspace$$

where *x*′(*y*) ​= ​0 because all points on the lateral axis have *x* ​= ​*x*_cross_ and we compute *z*′(*y*) deriving equation Eq. 21. The length of the leaf *L*_length_ is computed as

(24) $$L_{\text{length}}=\int_{{\hat{x}}_{\text{min}}}^{s_{l}}\sqrt{z^{\prime }{\left(x\right)}^{2}+y^{\prime }{\left(x\right)}^{2}}\;dx=\int_{{\hat{x}}_{\text{min}}}^{0}\alpha \;dx+\int_{0}^{s_{l}}\left(1-{tanh}^{2}\left(x\right)\right)\;dx,$$

where ${\hat{x}}_{\text{min}}$ is the resulting minimum value for *x* we got multiplying *x*_min_ for *s*_*l*_, *y*′(*x*) ​= ​0 because all points of the main axis have *y* ​= ​0, and we derived Eq. (19) and Eq. (20) to sum the length of the petiole and the length of the leaf blade. We show examples of the skeletons built with our approach in the supplementary material. One can make the skeletons more complex using different functions, or polynomials of higher grade to represent the axes. However, the results of our generative procedure suggest that our skeletons capture the characteristics of the used crop species.

## 5 Results and discussion

The main focus of this work is an approach to generate 3D leaf point clouds of known length and width. Using our data improves the performance of trait estimation approaches and enables a more fine-grained analysis of crop growth and productivity. We present our experiments to show the capabilities of our method and to support our key claims: (i) using our generated leaves $D_{\text{ours}}$ to tune trait estimation approaches perform better than using other generated leaf point clouds; and (ii) all generated leaves respect the leaf traits we condition on and have a high probability of being sampled from the real-world leaf distribution.

### 5.1 Experimental setup

**Datasets and Baselines:** We use the BonnBeetClouds3D [42] dataset, computed via photogrammetric reconstruction and bundle adjustement, and Pheno4D [43] dataset, captured with a laser scanning system. Both datasets provide single-leaf point clouds. We evaluate our approach by comparing our generated leaf point clouds $D_{\text{ours}}$ to the leaves generated by three possible *g* functions in our problem formulation in Eq. (3). First, a set of leaves generated using the procedural agriculture simulation software Helios [31] exported by means of a simulated LiDAR sensor, from now on called $D_{\text{H}}$. Second, we apply transformations specific to the agricultural domain from our previous work [44] to the leaves obtained from Helios to obtain a larger variety of leaves, that we call $D_{\text{HT}}$ where HT stands for “Helios ​+ ​transforms”. Third, we train LiDiff [45] to generate leaves conditioned on the skeletons using diffusion, from now on denoted as $D_{\text{LiDiff}}$. Lastly, we also use the real-world per-plot ground truth data $D_{\text{real}}$ to highlight the importance of per-leaf traits to improve the performance of the leaf trait estimation methods.

**Training Details and Hyperparameters:** We train our network using the Adam optimizer [46] with learning rate 0.001. In our loss, we set the weights of the different components to *λ*_0_ ​= ​1, *λ*_1_ ​= ​0.1, *λ*_2_ ​= ​0.1, *λ*_3_ ​= ​10, *λ*_4_ ​= ​0.01. These weights help preserve traits while producing realistic leaf point clouds. We use different scaling factors for the different plant species: $s_{l}∼U\left(0.02,0.50\right)$ and $s_{w}∼U\left(\frac{s_{l}}{4},s_{l}\right)$ for the sugar beets, $s_{l}∼U\left(0.15,0.90\right)$ and $s_{w}∼U\left(\frac{s_{l}}{10},\frac{s_{l}}{5}\right)$ for the maize and $s_{l}∼U\left(0.10,0.50\right)$ and $s_{w}∼U\left(\frac{s_{l}}{2},s_{l}\right)$ for the tomato leaves. We use *J* ​= ​2 for sugar beets and tomato leaves, and *J* ​= ​1 for maize leaves. We plan to make our code publicly available upon acceptance.

**Metrics:** We evaluate the estimated traits by comparing the mean and the standard deviation estimated by all approaches when trained on the different generated data. Additionally, we compute the Fréchet inception distance (FID) [47], the CLIP Maximum Mean Discrepancy (CMMD) [40], and the F-score computed by the precision (Pr) and recall (R) [41] explained in Sec. 4 .3 to estimate how close the distributions of the generated and real data are. We use the pre-trained networks from Mohammadi et al. [48] and Hedge et al. [39] to extract the embeddings **v**, both networks provide open-source code and pre-trained models. Embedding-based metrics, as the ones employed in our evaluation, are the standard approach to evaluate generative methods [41,[49], [50], [51], [52]]. These metrics provide a semantic and perceptually relevant comparison, allowing for distribution-level comparisons that would not be possible for distance metrics based on the raw points’ positions. To verify that we are not generating the same leaf when conditioned on one specific skeleton, we compute the mean and standard deviation of two different metric distances between multiple leaves $\hat{P}$ generated from the same skeleton input S.

### 5.2 Trait estimation

The first experiment evaluates how tuning off-the-shelf trait estimation approaches on $D_{\text{ours}}$ improves the performance compared to other datasets. We show that tuning on $D_{\text{ours}}$ provides better estimates in terms of mean and standard deviation without relying on costly manual annotations. We test the fine-tuned approaches on the validation set of BonnBeetClouds3D [42], which only provides mean and standard deviation per sub-areas – patches – of the field.

We use three trait estimation approaches *f*: (1) the approach by Choudhury et al. [12] fits a polynomial to the skeleton of the leaf and then computes the leaf length via integration; (2) the approach by Huang et al. [13] uses the principal components to define the direction of the length and width of the leaf and then computes the longest shortest geodesic distance along those directions via A∗ [53]; (3) Coherent point drift [14] uses GMMs to find the best correspondences between two set of points. Coherent point drift needs a source point cloud to deform, i.e., a leaf point cloud template, for which we use the leaf template defined by Marks et al. [15]. As explained in Sec. 4.1, this template mesh already defines the points belonging to the main and lateral axes, allowing us to compute the length and width of the leaf after the deformation carried out by Coherent Point Drift.

For $D_{\text{H}}$, $D_{\text{HT}}$, and $D_{\text{ours}}$, we have per-leaf traits, while $D_{\text{real}}$ only provides per-patch averages. This introduces a systematic error since all leaves from the same plot will have the same ground truth. For $D_{\text{LiDiff}}$ [45], we use our skeletons of known traits, without changing the noise generation and training procedure. We point out that, for our skeletons, we also know the ground truth leaf angle. However, since this was not in any dataset ground truth measurements, we were not able to use it for evaluation purposes.

**Fig. 4:**
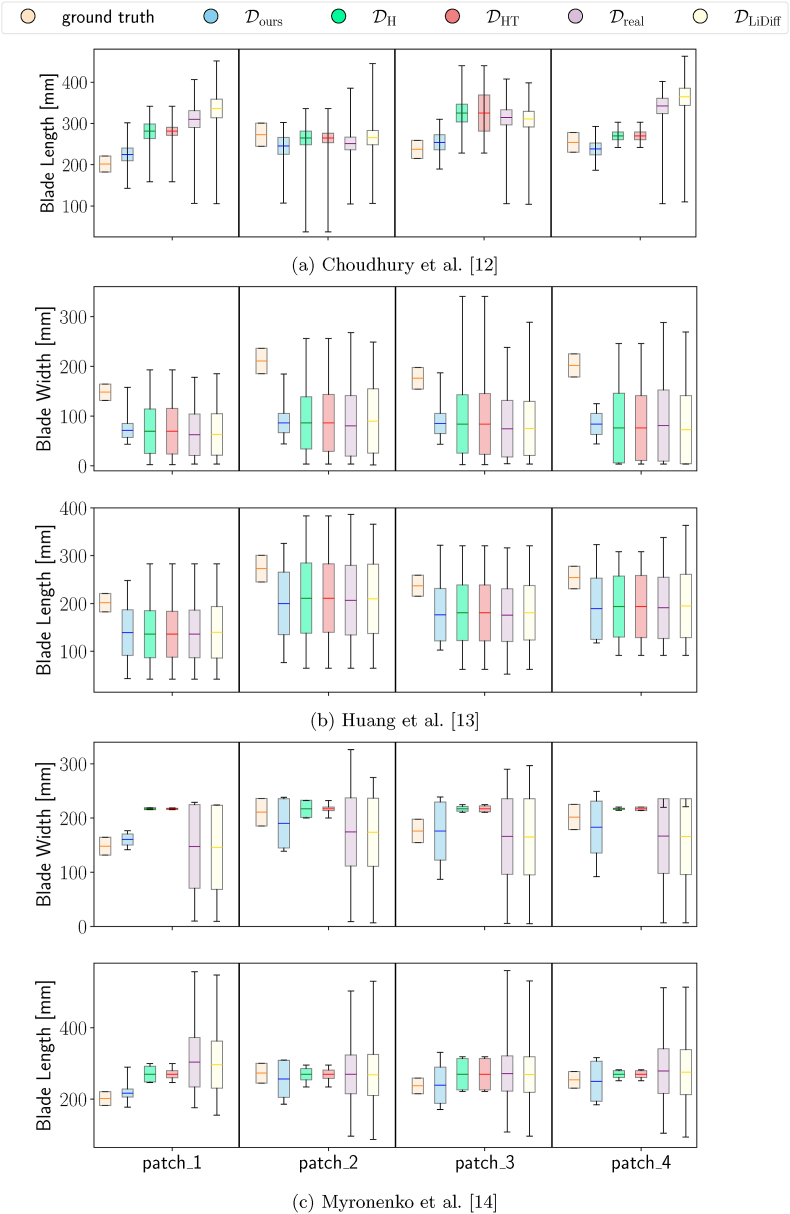
We show the leaf blade length and width estimated by the approaches for BonnBeetClouds3D [42] after tuning them on the different datasets. Each bar plot is centered on its mean, the size corresponds to its standard deviation, and we show the maximum and minimum estimated values.

In Fig. 4 (a), we show the results testing the approach by Choudhury et al. [12] on the validation patches of the BonnBeetClouds3D dataset. We see that the second patch is the one where tuning over $D_{\text{ours}}$ performs worse. This suggests that our generated point clouds do not align well with the leaves in this patch, which is the one with the larger leaves. This could also explain why we are the only one underestimating the size of patch_4, where using $D_{\text{H}}$ and $D_{\text{HT}}$ results in smaller variances and better maximum and minimum estimates compared to the other patches. In general, the maximum and minimum estimates obtained tuning on $D_{\text{ours}}$ are better, even when other datasets provide a mean closer to the ground truth.

The results of Huang et al. [13] shown in Fig. 4 (b) provide similar estimates across all the patches and datasets, suggesting an algorithmic limitation rather than dataset influence. The pipeline has few hyperparameters to remove outliers and define the path cost of the A∗ algorithm [53] used to compute the leaf blade length and width. We think that the PCA-based method struggles with the complex heart shape of the sugar beet leaf and with occlusions, misidentifying the main axis and, thus, leading to estimate errors. Failures to identify the main axis would also explain the large differences in maximum and minimum estimates. The differences in the results likely depend on the dataset's resolution and sparsity, which would impact the outliers detection and the computation of the distances.

We show in Fig. 4 (c) tuning Coherent point drift [14] on $D_{\text{ours}}$ provides means closer to the ground truth but with larger standard deviations. The second patch remains problematic, confirming the trend observed for the approach by Choudhury et al. [12]. While $D_{\text{H}}$ and $D_{\text{HT}}$ perform better on patch_2, the uniformity of their results suggests a potential overfitting or a failure to capture the data diversity. Tuning the approaches on $D_{\text{real}}$ yields diverse results but large standard deviations, especially for the blade width, likely because of the lack of per-leaf ground truths. Similarly, also using $D_{\text{LiDiff}}$ shows high standard deviations, likely because the generation procedure does not preserve the traits accurately. More details about the data generated with LiDiff can be found in the supplementary material.

The results show that using accurate per-leaf traits, even when artificially generated, improves the estimation results on real-world leaves. Our approach enables precise trait estimation without manual labeling or expensive expert knowledge. This is crucial for breeders and agronomists assessing plant traits linked to crop growth and productivity. However, ambiguity in defining leaf width (e.g., midsection vs. widest point) complicates evaluation. Mismatches in width definitions across datasets, generative models, and estimation methods introduce systematic errors, highlighting the need for standardization in trait measurement.

### 5.3 Realistic data generation

The second set of experiments assesses how closely our generated leaf point clouds match real-world distributions. We demonstrate that our approach generates leaf point clouds with features similar to real-world data, making them valuable for tuning trait estimation approaches to use in real-world scenarios. Additionally, our method generated diverse leaves while maintaining specified blade length and width, bridging the gap between simulated and real-world data. As detailed in Sec. 5.1, we evaluate the generated points clouds with the metrics explained in Sec. 4.3. They compare distributions of embeddings, which we extract using the two different pre-trained networks mentioned in Sec. 5.1.

#### 5.3.1 Leaf distribution

**Figure 5:**
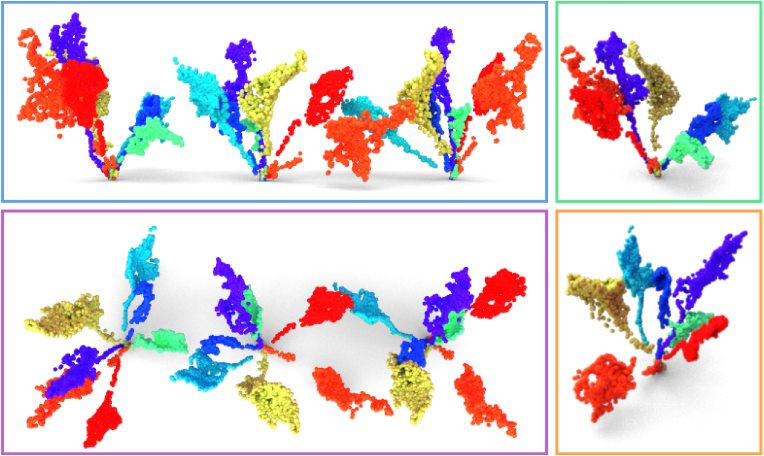
Examples of sugar beet leaves generated by our approach with different leaf angles, stem lengths, and blade lengths and widths. We show a side (blue rectangle) and a top view (purple rectangle) of three plants generated using the same leaves with different orientations and positions. We show zoomed in views of the first (green rectangle) and second plant (orange rectangle).

We use the validation set from BonnBeetClouds3D, from now on called SugarBeets dataset, and the unlabeled plants from Pheno4D as real-world target point clouds. We show examples of the leaves generated by our approach trained on SugarBeets in Fig. 5, where we use all the information we have thanks to the skeleton to also merge our generated leaves into complete plants. We can see that the network learned different leaf shapes that are not uniquely connected to the leaf dimensions. For example, the yellow and green leaves have a similar shape even if the green leaf is smaller. We can also modify the stem angle, giving the desired orientation to each leaf; this is clear for the orange leaf whose is almost vertical in the left plant and almost horizontal in the central plant. Looking at the light blue leaf, we can see that we are also able to rotate the leaf around the stem axis, thus changing the surface orientation. We compute all metrics for $D_{\text{ours}}$, $D_{\text{H}}$, $D_{\text{HT}}$ and $D_{\text{LiDiff}}$. Since LiDiff requires conditioning on skeletons but does not provide a skeleton generation procedure, we use the skeletons of the training set to generate new leaves, potentially giving it an advantage over methods relying on domain expertise or procedurally generated skeletons. Table 1 shows the results of the CMMD, FID, and F-score. For the F-score, we use both feature extractors, i.e., the network by Mohammadi et al. [48] and by Hedge et al. [39], to isolate network-specific influences. Fitting a Gaussian on the CLIP embeddings of the real-world point clouds was failing and starting with non-default initializations provided inconsistent results, thus we do not include the FID metric with CLIP embeddings. Since the improved precision and recall metrics depend on the number of neighbors used to construct the real and generated data manifolds (Φ_*r*_ and Φ_*f*_), we evaluate the F-score across multiple values of *k*, specifically *k* ​∈ ​{2, 4, 8, 16, 32, 48, 64, 96}. We then report the mean F-score over these values to provide a more stable and robust estimate.

**Table 1:** Evaluation of the Fréchet inception distance (FID), F-score, and CLIP Maximum Mean Discrepancy (CMMD) for the leaf point clouds generated by the different approaches compared to the test sets. Our approach outperforms the others on most metrics across the different datasets. Our results are highlighted using gray colored rows.

Dataset	Generated Data	FID *↓*	F-score *↑*	F-score ​+ ​CLIP *↑*	CMMD *↓*
SugarBeets	$D_{\text{H}}$	312.84	0.01	0.01	169.71
	$D_{\text{HT}}$	14.01	**0.36**	0.01	28.45
	$D_{\text{LiDiff}}$	26.89	0.35	0.11	22.05
	$D_{\text{ours},\text{rec}}$	108.73	0.03	0.17	20.46
	$D_{\text{ours}}$	**13.71**	0.21	**0.20**	**19.53**
Maize	$D_{\text{H}}$	11.28	0.19	0.02	29.39
	$D_{\text{HT}}$	0.17	0.39	0.17	16.36
	$D_{\text{LiDiff}}$	1.05	0.22	0.09	65.14
	$D_{\text{ours}}$	**0.12**	**0.55**	**0.36**	**11.51**
Tomato	$D_{\text{H}}$	7.89	0.01	0.06	28.26
	$D_{\text{HT}}$	5.29	0.02	0.06	24.16
	$D_{\text{LiDiff}}$	6.66	0.05	0.10	65.42
	$D_{\text{ours}}$	**2.76**	**0.15**	**0.17**	**12.39**

We see that applying our domain-specific transforms to $D_{\text{H}}$ improves all metrics across all datasets. The results are generally better on Pheno4D, likely because they provide leaves at different growth stages while the SugarBeets dataset was recorded over the same day. Overfitting to the exact growth stage could lead to a boost, explaining the results of LiDiff which uses the skeleton of the training point clouds. Our approach outperforms the others across most of the investigated scenarios, except for FID ​+ ​CLIP and the F-score on the SugarBeets dataset, where the feature extractors yield conflicting results. For the SugarBeets dataset we also provide an ablation study on our approach, namely $D_{\text{ours,rec}}$, where we keep everything the same but we train using only the reconstruction losses, without the distribution ones. We can see that using the distribution losses improves all metrics, especially the FID and the F-score that get 7 times better. This shows the contribution of our distribution loss functions and the importance of a distribution supervision while generating the leaf point clouds.

Given the low F-score values in Tab. ​1, we compute the realism score as in Eq. ​18 and re-evaluate the generative approaches on the SugarBeets dataset, where the two feature extractors contradict each other. We noticed that the number of samples filtered out by the realism score was high, especially for low values of *k*. Table 2 shows the F-score filtering out elements with low realism and considering only results where more than half of the generated leaves were used. Most results improve while increasing the minimum realism score, but many approaches fail when the minimum accepted realism is too high. When realism exceeds 1.0, only our generated leaves consistently allow F-score computation with both models, indicating strong alignment with real-world distributions. This explains why tuning on our data enhances leaf trait estimation. However, the feature extractors still disagree, highlighting the need for a standardized feature extractor, as it exists in the image domain, or even a domain-specific feature extractor that better captures important features in the agricultural domain.

**Table 2:** F-score computed for all the approaches using different values of realism to filter out the outliers. When less than half of the samples were valid, we do not report any result (−). The more samples we filter out, the higher the metrics. Our approach is the only one that always provides enough samples in the dense area of the distribution.

Realism	Generated Data	F-score *↑*	F-score ​+ ​CLIP *↑*
0.0	$D_{\text{H}}$	0.01	0.01
	$D_{\text{HT}}$	**0.36**	0.01
	$D_{\text{LiDiff}}$	0.35	0.11
	$D_{\text{ours}}$	0.21	**0.20**
0.5	$D_{\text{H}}$	0.01	0.01
	$D_{\text{HT}}$	**0.48**	0.02
	$D_{\text{LiDiff}}$	0.33	0.11
	$D_{\text{ours}}$	0.22	**0.23**
1.0	$D_{\text{H}}$	0.02	0.02
	$D_{\text{HT}}$	0.45	0.04
	$D_{\text{LiDiff}}$	**0.64**	0.11
	$D_{\text{ours}}$	0.29	**0.55**
1.5	$D_{\text{H}}$	–	–
	$D_{\text{HT}}$	**0.80**	–
	$D_{\text{LiDiff}}$	–	–
	$D_{\text{ours}}$	0.29	**0.90**

#### 5.3.2 Leaf variety

**Fig. 6:**
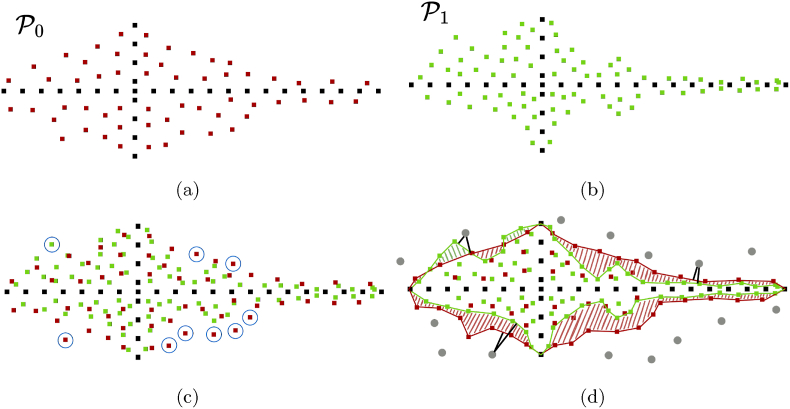
(a) and (b) are two leaves generated from the same skeleton. We show in (c) that when using the Chamfer distance, the outliers in the blue circles are the only ones providing a meaningful distance since most of the points are in the same area. In (d), we show our proposed point-to-mesh distance, where we compute the difference between the distances from each gray point to the two meshes.

Our method generates leaf point clouds from skeletons, but we want to ensure diversity by generating different leaves given the same skeleton. This enhances the dataset variety without altering skeleton-building procedures or training multiple generative networks *g*. A diverse dataset is crucial to optimize trait estimation approaches avoiding overfitting to common samples. In this experiment, we input the same skeleton multiple times and compare the generated leaves by computing two distances. First, we use the Chamfer distance. Second, we compute the meshes from the leaf point clouds via ball pivoting [54] and measure the surface differences as the differences of the distances from the meshes to randomly sampled 3D points. In Fig. 6, we show a simplified 2D example of the point-to-mesh distance.

In Table 3, we report the mean chamfer and point-to-mesh distances on 10 leaves generated with the same skeleton averaged over 10 runs. Since we cannot condition the Helios software on a skeleton, we report the results only for our approach and LiDiff. While the chamfer distances have similar means, LiDiff's standard deviation is approx. 4.5 times higher, likely due to the weaker compliance to the skeleton. For the point-to-mesh distance, we see a larger difference in the mean, more than 1 ​cm, and again the standard deviation of LiDiff is more than 3 times ours.

**Table 3:** Average mean and standard deviation for the chamfer and point-to-mesh distances, computed over 10 trials on 10 leaves generated conditioning the network with the same skeleton.

Generated Data	Chamfer [mm]		point-to-mesh [mm]	
	mean	std	mean	std
$D_{\text{LiDiff}}$	6.14	7.53	55.82	57.87
$D_{\text{ours}}$	5.69	1.69	67.69	17.58

Given our previously reported results, we think that our approach provides more precise per-leaf ground truths for tuning trait estimation methods. In contrast, LiDiff produces a wider variety of leaves, at the cost of higher leaf trait errors. Since LiDiff is a general-purpose approach for conditioned diffusion, adding specific losses could help the approach follow more closely the input skeleton and improve the results.

## 6 Limitations and future work

In this article, we tested our approach on different crop varieties, all exhibiting similar shape complexity. Our approach works also on more complex shapes, as compound leaves – leaves where the blade is divided into two or more leaflets, if enough data is provided. Since the network learns from real-world data, we can use the whole compound leaf as it is for the network to learn its shape. Nonetheless, structural complexity presents additional challenges, and some enhancements could improve the convergence speed and the overall performance. One improvement involves modifying the algorithm for building the skeleton. For example, we could combine multiple skeletons to capture the morphology of a compound leaf. Additionally, adjusting the number of Gaussians *J* in the GMM can improve the initial position of the points, leading to more efficient training. While these changes are not strictly required, they could reduce the need for training data and provide a better a more effective initialization. Another potential direction is to learn the shape of single leaflets instead of the entire compound leaf. This method wouldn't require algorithmic changes, but it would require access to single leaflet point clouds – harder to obtain than single leaves. Furthermore, knowledge about the leaf structure would be needed to reconstruct complete leaves from the generated leaflets.

As with any deep-learning method, our approach assumes that the distributions of the training and inference data is similar. When there is a mismatch – such as training on simple low-resolution skeletons and then performing inference on complex and high-resolution skeletons, or vice versa – the performance may degrade. However, this is the only assumption we make on the skeletons. As demonstrated in our experiments, our methods works with different skeletons extracted by different methods without requiring adaptation.

For future work, we aim to evaluate our approach on more fine-grained tasks, such as generating distinct varieties within a single crop species. This would require large and variety-specific datasets, as the network must learn finer details. Currently, the primary limitation for this direction is the lack of available data. Moreover, existing evaluation metrics often rely on networks pre-trained on large datasets of common objects [55,56], which may struggle to differentiate between single crop species varieties. A domain-specific foundation model tailored to plant data would yield more reliable evaluations. Another possible direction for future research is integrating our method with plant growth models. Since our network can be trained on leaves from specific growth stages, we can generate a large variety of leaves for each stage. Plant growth models could provide the expected leaf blade length and width based on environmental and nutritional input, allowing for more realistic simulations without the need for external user-defined inputs.

## 7 Conclusions

In this paper, we address the data bottleneck when working with 3D point clouds of leaves and presented an approach that can generate realistic leaf point clouds with given traits, such as leaf length and width. Our method generates realistic leaves that can then be directly used to train or fine-tune off-the-shelf leaf trait estimation approaches. We have shown that optimizing the hyperparameters of different methods on our generated data achieves better results than using per-plot ground truth averages or leaves generated by other state-of-the-art approaches. Using our data allows for more precise and fine-grained estimation of traits that directly influence crop growth and productivity. Our results suggest that per-leaf ground truth data is essential for estimating leaf traits and that generated data can significantly boost the performance of existing methods. We run experiments on three different plant species, demonstrating the potential of learning from real-world data without requiring labels. Even when real-world per-leaf ground truth measurements are available, our approach can generate leaves of different lengths and widths to fill potential gaps in the collected data. Thus, we can obtain unbiased datasets from different growth stages, reducing the efforts of the experts and the need for destructive measurements.

## Author contributions

The authors confirm contribution to the paper as follow: study conception and design: G. Roggiolani; interpretation of results: G. Roggiolani; draft manuscript: G. Roggiolani, Brian. N. Bailey, J. Behley, C. Stachniss; funding acquisition and project coordination: C. Stachniss. All authors reviewed the results and approved the final version of the manuscript.

## Funding

This work has partially been funded by the 10.13039/501100001659Deutsche Forschungsgemeinschaft (DFG, German Research Foundation) under Germany's Excellence Strategy, EXC-2070 – 390732324 – PhenoRob.

## Data availability

We use two publicly available datasets. Pheno4D [43] is available at the url: https://www.ipb.uni-bonn.de/data/pheno4d/index.html. It contains maize and tomato plants measured daily, over 12 and 20 days respectively. Only half of the point cloud are labeled with temporally consistent labels. The SugarBeets dataset BonnBeetClouds3D [42], available at https://bonnbeetclouds3d.ipb.uni-bonn.de/, and it contains point clouds from a 1.5 ​m by 7 ​m field plot. The training set consists of 128 plants, with a total of 1782 leaves; the validation set consists of 17 plants, with a total of 260 leaves. The dataset provides labels for single plants and leaves, and the key points of the leaf base, tip, right and left corner, for each labeled leaf. The leaf blade length and width, together with the petiole length and width, are given per plot, where a plot is a 1 ​m by 1 ​m patch of the field.

## Declaration of competing interest

The authors declare that they have no known competing financial interests or personal relationships that could have appeared to influence the work reported in this paper.
