# Supplementary material for: Generation of labeled leaf point clouds for plants trait estimation
Source: Plant Phenomics. 2025 Jun 16;7(3):100071. doi: 10.1016/j.plaphe.2025.100071 (PMC12710001; doi:10.1016/j.plaphe.2025.100071)
Supplement: Multimedia component 1 [file mmc1.pdf]

Generation of Labeled Leaf Point Clouds for Plants Trait Estimation

Gianmarco Roggiolani[1*], Brian N. Bailey[2], Jens Behley[1], and Cyrill Stachniss[1,3]

[*]Address correspondance to: groggiol@uni-bonn.de
[1]Center for Robotics, University of Bonn, Germany
[2]Department of Plant Science, University of Davis, United States
[3]Lamarr Institute for Machine Learning and Artificial Intelligence, Germany

Abstract

Today, leaf trait estimation remains a labor-intensive process. The effort to obtain ground truth measurements limits how accurately this task can be performed automatically. Traditionally, plant scientists manually measure the traits of harvested leaves and associate them with sensor data, which is key for training machine learning approaches and to automate the processes. In this paper, we propose a neural network-based method to generate synthetic 3D point clouds of leaves with their associated traits to support approaches for phenotyping. We use real-world leaf point clouds to learn how to generate realistic leaves from a leaf skeleton, which is automatically extracted. We use the generated leaves to fine-tune different leaf trait estimation methods. We evaluate our generated data using different trait estimation methods and compare the results to using real-world data or other synthetic datasets from agricultural simulation software. Experiments show that our approach generates leaf point clouds with high similarity to real-world leaves. Tuning trait estimation methods on our generated data improves their performance in the estimation of real-world leaves' traits, making our data crucial for developing and testing data-driven trait estimation methods. Accurate trait estimation is key to understanding crop growth, productivity, and pest resistance, as leaf size directly influences photosynthesis, yield potential, and vulnerability to insects and fungal growth.

1 Introduction

The global demand for food, fuel, and fiber constantly increases due to the growing world population. Agricultural systems must meet this growing demand while producing resources more sustainably. The common practice of expanding farmland has reached its limit due to desertification, salinization, and soil erosion. The problem of increasing crop production is tackled by developing new crop varieties, with higher crop yield and toward resistance to stress and diseases [1–3]. Prior works [4–6] have linked crop productivity to different traits of the leaf morphology, such as the leaf width and length or its shape.

Phenotyping is the process of measuring traits of plants. Today, this task is still mainly performed by workers measuring observable traits manually, making it an expensive, time-consuming, and difficult-to-scale task [7]. This limitation is evident in agricultural datasets, which often provide only the average traits computed over a few manually selected leaves, reducing the granularity and accuracy of successive analyses. However, this is often not enough to evaluate approaches that aim to estimate per-plant traits and is even more problematic for training deep learning approaches, which usually require large amounts of labeled data for supervised training [8]. Obtaining such data is time-consuming, costly, and often a bottleneck for algorithm development.

In this paper, we tackle the problem of the lack of training data by developing a generative approach to produce leaf point clouds with a given length and width that we use to optimize approaches for the estimation of these leaf traits. Prior works [9–11] on trait estimation focused on leaf instance segmentation on images treating the number of leaves as the main trait. However, images provide a limited understanding of angles and curvatures, which are needed to estimate the length and width of bending leaves. 3D point clouds can better capture the geometry of the leaves, allowing for more accurate estimation of geometric leaf traits, such as the leaf width and length. Most of the approaches for 3D data are rule-based [12–15] instead of data-driven [16, 17] since the lack of data with reference traits does not allow for training of learning-based methods to estimate traits different from the number of leaves. However, such approaches still need fine-tuning to achieve satisfactory performance.

The main contribution of this paper is a novel approach for generating leaf point clouds with their associated leaf traits. Our work opens the road to the development, benchmarking, and comparison of next-generation trait estimation techniques previously limited by the lack of data. Unlike the traditional template leaf model – a mechanistic representation developed by expert plant scientists to capture the leaf morphology, we use a generative network trained on real-world data. As input, our network receives a point cloud representing a leaf skeleton with its traits as high-level descriptors. The network generates realistic point clouds of leaves as output. Training on real-world data allows us to generate leaves with distributions similar to real ones, without the need for additional expert knowledge for each different plant species. We generate new leaves by providing a skeleton of the desired length and width. In this article, we decompose the problem of trait estimation into two parts: firstly, a generative method produces leaf point clouds with their respective leaf width and length, and secondly, we use the generated data to optimize the parameters of a trait estimation approach. Further details on the problem decomposition can be found in the supplementary material. We compare our approach against other geometric and learning-based leaf generation methods, showing that our generated leaf point clouds are similar to the real-world leaf distribution. Then, since our approach tackles the problem of the lack of data for trait estimation methods, we show that tuning different off-the-shelf trait estimation approaches on our generated data significantly improves the accuracy and precision of real-world leaf trait estimation. We evaluate our approach on multiple datasets of different crop species. In sum, we make two key claims: (i) using our generated leaves to tune trait estimation approaches performs better than using other generated or real-world leaf point clouds; and (ii) all generated leaf point clouds respect the leaf traits we condition on and have a high probability of being sampled from the real-world leaf distribution. We plan to make our code

publicly available to enable further development of trait estimation methods.

2 Related Work

The problem of trait estimation in agriculture is still largely tackled by manual measurements performed by domain experts. This process is expensive, labor-intensive, and prone to introduce biases in the collected data. For example, bigger leaves are easier to identify and remove, leading to them being over-represented and resulting in a biased estimate of the trait distributions. As with many other agricultural tasks, trait estimation can be automatized using robotic platforms equipped with perception systems [18–20]. These systems capture the leaf data and analyze it without human intervention at a fine-grained scale and in a non-destructive fashion, i.e., without the need to remove the leaf from the plant.

2D Trait Estimation: Lately, several computer vision approaches have been developed to estimate phenotypic and functional traits from 2D images. Most of them focused on leaf segmentation and counting, initially using heuristic approaches and later based on neural networks. Multiple geometric segmentation techniques have been employed to segment single leaves, such as the region growing algorithm in the work by Pape et al. [21], adaptive thresholding in the work by Bai et al. [22], and the Sobel operator used by Wang et al. [23]. Heuristic approaches often rely on tuning several hyperparameters to obtain satisfactory performance. Deep learning methods typically require fewer manually tuned parameters, as their weights are optimized during training with labeled data. Deep learning approaches using convolutional neural networks have been shown to outperform heuristic methods for leaf segmentation, especially when they exploit knowledge about the plant structure [10, 24]. However, these approaches only count the number of leaves or estimate the leaf area [25]. Because of the projective view of the images, it is challenging for any image-based approach to estimate per-leaf traits, such as the leaf length and width, which provide more information about crop growth and pest resistance.

3D Trait Estimation: Using 3D data, i.e., point clouds or meshes, allows for estimating more complex leaf traits, such as widths and lengths of the leaves, which are hard to determine using images only. The potential of 3D data for plant trait estimation has already been shown by several approaches developed in the context of horticulture [26, 27], where automatic estimation of the size and color of single fruits is crucial for automatic harvesting and yield estimation. In the context of leaf trait estimation, many works still focus on controlled environments [12, 13], assuming perfect 3D data, with only one plant in the scene, and thus, with little to no occlusion. The problem of handling data that is not fully visible because of occlusions or missing viewpoints severely impacts the ability to segment the leaves and estimate their widths and lengths. This limits the application of such methods in complex real-world scenes.

Marks et al. [15] tackle this limitation by means of template reconstructions. They propose using a deformable template mesh to reconstruct the leaves, even in the case of missing parts due to self-occlusions or occlusions from other plants. Once the leaf is reconstructed in 3D without missing parts, they estimate the position of the leaf center, tip, and right and left corners. The majority of the approaches use the geometric structure of the data and the knowledge about the appearance of

the plants to segment the leaves and estimate the relevant traits. The lack of ground truth traits per single leaf limits the deployment of deep-learning approaches. Recent generative approaches could solve this problem by producing synthetic annotated data for supervised learning.

Generated Data for the Agricultural Domain: In recent years, several works have proposed to use artificially generated data in the agricultural domain. Most of these approaches generate images to train networks for crop-weed segmentation [28, 29]. In the context of 3D data generation, Helmrich et al. [30] propose a pipeline requiring user-defined parameters for the plant, the root system, and the functional model of the plants. This makes the approach suitable for different functional and structural analyses but requires expert knowledge for modeling the plant and setting parameters. Additionally, they generate the 3D agricultural scene only to export images of the scene. The work by Bailey [31] generates synthetic fields of different crop species. Their simulation software focuses on functional traits, i.e., radiation, photosynthesis, and water conduction. Similarly, expert knowledge is required to build the plant and set the parameters for each crop species. None of the works can exploit real-world data collected automatically, for example, by a robot or mobile sensing system. Instead, they require access to manually measured traits to set the parameters.

Our work falls into the category of methods that generate data for the agricultural domain. We propose an approach to automatically generate point clouds of leaves with known widths and lengths. In line with this methodological approach, Choi et al. [32] use the 3D Plant simulator Helios [31] to simulate a 3D agricultural scene from which they create a synthetic image dataset for training networks. As Helmrich et al. [30], they build 3D scenes only to export images of the scene as a dataset for training deep-learning methods. Our main contribution is the generation of leaf point clouds annotated for leaf trait estimation without the requirement for labeled data or expertise that can be learned from real-world data in an unsupervised fashion. We also show how directly using our generated point clouds enables more accurate leaf trait estimation for real-world leaves.

3 Problem Formulation

We formally define the problem before explaining our proposed method for generating point clouds of leaves. Leaf trait estimation is performed by a method that we express as a function

$$f(\mathcal{P}_i, \boldsymbol{\theta}) = \hat{\mathbf{t}}_i, \tag{1}$$

where $\mathcal{P}_i$ is the input leaf point cloud, $\boldsymbol{\theta}$ are the approach's parameters, $\hat{\mathbf{t}}_i \in \mathbb{R}^T$ is the vector of T estimated traits for the input leaf $\mathcal{P}_i$, each one a scalar. We can find the optimal parameters of the approach given a dataset of D leaf point clouds with traits $\mathcal{D} = \{(\mathcal{P}_i, \mathbf{t}_i)\}_{i=1}^{D}$ as

$$\boldsymbol{\theta}^*_{\mathcal{D}} = \arg\min_{\boldsymbol{\theta}\in\Theta} \sum_{(\mathcal{P}_i,\mathbf{t}_i)\in\mathcal{D}} e\left(f(\mathcal{P}_i, \boldsymbol{\theta}),\, \mathbf{t}_i\right) = \arg\min_{\boldsymbol{\theta}\in\Theta} \sum_{\mathbf{t}_i\in\mathcal{D}} e\left(\hat{\mathbf{t}}_i,\, \mathbf{t}_i\right), \tag{2}$$

where Θ is the set of possible parameters $\boldsymbol{\theta}$, and $e\left(\hat{\mathbf{t}}_i,\, \mathbf{t}_i\right)$ is a function computing the error between the estimated traits $\hat{\mathbf{t}}_i$ and the ground truth traits $\mathbf{t}_i$ in $\mathcal{D}$. The error function e used may depend on the estimated traits, e.g., the cosine similarity is appropriate for the angle between the leaf and

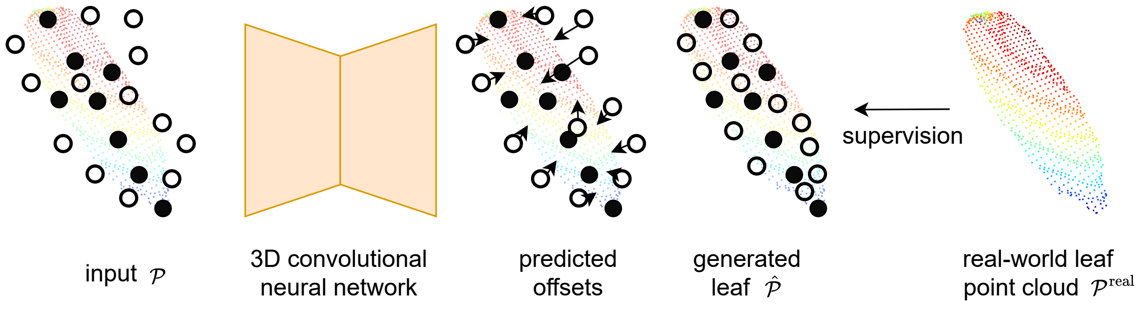

Figure 1: Overview of our approach. We show the input point cloud $\mathcal{P}$ made up from the skeleton points in and in white hte points sampled from the GMM. Our network predicts per-point offsets depicted as arrows. Adding the offsets to the points' positions we obtain $\mathcal{P}$. We supervise the network using real leaf point clouds $\mathcal{P}^{\text{real}}$.

the plant stem but not for the length of the leaf blade. As for any optimization procedure, the final performance of the trait estimation approach f depends on the dataset's completeness and reliability. As already mentioned in Sec. 1 , the real-world agricultural datasets $\mathcal{D}_{\text{real}}$ associates multiple leaf point clouds $\mathcal{P}_i^{\text{real}}$ with the same average traits computed over a few manually selected leaves. We want to tackle the problem of *generating a dataset* with known traits *for each* leaf point cloud. We introduce the generative problem as defining a function

$$g(\mathbf{t}_i) = \hat{\mathcal{P}}_i, \tag{3}$$

that generates leaf point cloud $\hat{\mathcal{P}}_i$ for given traits $\mathbf{t}_i$. In this way, we can generate a new dataset

$$\mathcal{D}_g = \{(g(\mathbf{t}_i), \mathbf{t}_i)\}_{i=1}^{D} . \tag{4}$$

This new dataset $\mathcal{D}_g$, with per-leaf ground truth traits $\mathbf{t}_i$ is used to find the best parameters $\boldsymbol{\theta}^*_{\mathcal{D}_g}$ for any given trait estimation method f. The generative function $g(\mathbf{t})$ must generate realistic data to obtain a valuable dataset and, thus, parameters $\boldsymbol{\theta}^*_{\mathcal{D}_g}$ that perform well on real-world point clouds.

4 Materials and Methods

We propose a novel approach that generates synthetic leaf point clouds $\hat{\mathcal{P}}$ with known traits. Instead of relying on a mechanistic model, we train a 3D convolutional neural network to generate synthetic leaf point clouds of desired traits $\mathbf{t}$. This is the g function of our problem formulation in Eq. (3). An overview of our approach is shown for an exemplary tomato leaf in Fig. 1.

In our work, we consider the leaf blade length and width as traits. Such traits are intrinsic in the leaf skeleton point cloud extracted from real-world leaves. We do not need their actual values to train our network. In Sec. 4 .1, we illustrate how to obtain the skeleton point clouds $\mathcal{S}$ from the point clouds of real leaves $\mathcal{P}^{\text{real}}$. We do not pose constraints on how to acquire the real-world point

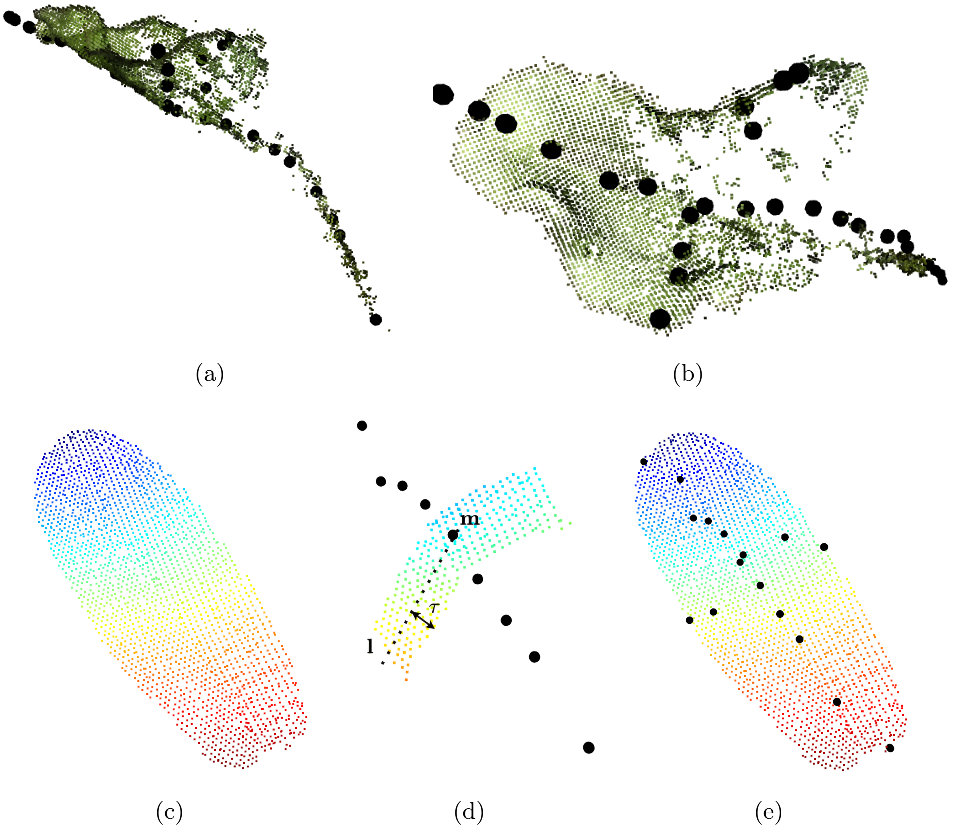

Figure 2: We show the extracted skeleton using the approach by Marks et al. [15] (top) for a sugar beet leaf, and the approach by Magistri et al. [34] (bottom) with our adaptation for a maize leaf. The skeleton $\mathcal{S}$ is always shown in black circles. We show the view from the side (a) and the top (b). For the maize leaf in (c), we show the skeleton extracted along the main axis and the points of the leaf slice cut around $\mathbf{m}$ in (d). In (e), the final skeleton with main and lateral axes.

clouds. The datasets we use have either been created by means of a laser scanning system with sub-millimeter accuracy or using photogrammetric reconstruction including bundle adjustment [33] on a set of images of the field. Then, in Sec. 4 .2, we describe how we add more points to the skeleton point cloud $\mathcal{S}$ to capture the shape of the whole leaf and obtain the input $\mathcal{P}$ for our network. We then explain the network's architecture. In Sec. 4 .3, we explain the loss we minimize during our training. Sec. 4 .4 describes how we build skeletons and compute accurate traits since we know the functions and limits that define the skeleton. Our network uses these skeletons to generate new leaf point clouds of known leaf blade length and width.

4 .1 Extraction of Leaves Skeletons

The first step of our approach extracts skeleton point clouds $\mathcal{S}$ of real-world leaves $\mathcal{P}^{\text{real}}$. We use two existing approaches by Marks et al. [15] and by Magistri et al. [34] to show that our approach can work with different skeleton extraction methods. The skeleton serves as a structural backbone

of the leaf, capturing the petiole, the main axis along the leaf length, and the lateral axis along the leaf width. In the literature, there is no universal definition of leaf width. For the approach by Marks et al. they define it on their template, while for the approach by Magistri et al., we define it as the width at the midsection of the leaf.

Marks et al. [15] manually define all the points and faces for a template mesh of a leaf that they deform to fit it to real leaf point clouds $\mathcal{P}^{\text{real}}$. They also define which points in the template represent the center, tip, right and left corners of the leaf, and which subsets of points represent the main axis, the lateral axis, and the petiole. The template targets sugar beet plants and new template meshes are needed for each new crop species. Since they define the points in the template belonging to the main and lateral axis, after fitting the template mesh to the leaf point cloud $\mathcal{P}^{\text{real}}$, we use the positions of such points as points for our skeleton point cloud $\mathcal{S}$. The top row of Fig. 2 shows the extracted skeleton for one exemplary sugar beet leaf.

We use the approach by Magistri et al. [34] to extract the skeleton point clouds $\mathcal{S}$ of leaves of tomato and maize plants. The main limitation of their approach is that it only provides the points of the skeleton along the main axis of the leaf, which usually represents the leaf length. Thus, their approach does not detect the points of the lateral axis, i.e., along the width direction of the leaf. Magistri et al. [34] generate a chain of n 3D points and fit it to the leaf point cloud. In the bottom row of Fig. 2, we show how to use their approach to also compute the points of the skeleton along the width direction of the leaf. After computing the n points of $\mathcal{S}$ along the main axis, we compute the median point $\mathbf{m} \in \mathbb{R}^3$, and the direction $\mathbf{n}$ of the lateral axis of the leaf as the second principal component extracted using principal component analysis on $\mathcal{P}^{\text{real}}$. We then cut a slice of the leaf around $\mathbf{m}$, preserving all points in the direction of $\mathbf{n}$ and removing points whose distance from the line $\mathbf{l} = \mathbf{m} + c\mathbf{n}$, where $c \in \mathbb{R}$, is larger than τ. This slice represents the central section of the leaf, from which we want to extract the points representing its width. In Fig. 2 (d), we show the skeleton along the main axis over the points that we keep at the end of this step. We then apply the approach only on the points in the area of interest to detect the skeleton points in the direction of the leaf width. The final result, which we obtain by combining the points from this two-step approach, is shown in Fig. 2 (e).

4 .2 From Skeletons to Network Outputs

We generate the leaves using a neural network, specifically a 3D U-Net [35] based on KPConv [36]. At the end of the previous section, we obtained the skeleton point cloud $\mathcal{S}$ with $\tilde{N}$ points $\mathbf{P}_{\text{skeleton}} \in \mathbb{R}^{\tilde{N}\times 3}$ representing the leaf skeleton. To reconstruct a complete leaf $\mathcal{Y}$, we add extra points beyond those of the skeleton to have enough points to ensure a realistic shape. We call N the total number of points in the point cloud $\mathcal{P}$ that we use as input for the network. We set $N = \tilde{N} + \delta\tilde{N}$, where $\delta \in \mathbb{Z}^+$ is a parameter that scales the number of total points according to the number of points in the skeleton $\mathcal{S}$. We sample the extra points $\mathbf{P}_{\text{sampled}} \in \mathbb{R}^{\delta\tilde{N}\times 3}$ from a Gaussian mixture model (GMM) [37] fitted to the original skeleton points $\mathbf{P}_{\text{skeleton}}$. A GMM is a probability distribution of density

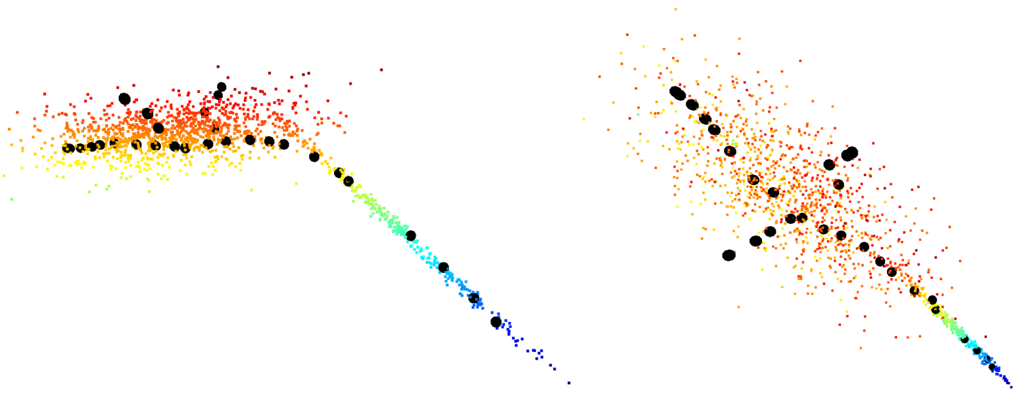

(a) (b)

Figure 3: We show the point cloud $\mathcal{P}$, i.e., the input of our generative function g. The skeleton points are shown in black circles. The other points are sampled from the Gaussian Mixture Model fitted on the skeleton. We show the view from the side (a) and the top (b).

$$p(\mathbf{p}_{\text{sampled},u}) = \sum_{j=1}^{J} \pi_j \, \mathcal{N}\left(\mathbf{p}_{\text{sampled},u}; \boldsymbol{\mu}_j, \boldsymbol{\Sigma}_j\right), \tag{5}$$

where $\mathbf{p}_{\text{sampled},u} \in \mathbb{R}^3$ is the position of the $u-$th sampled 3D point, J is the number of distributions in the mixture, π_j is the probability of selecting the $j-$ th distribution, $\boldsymbol{\mu}_j \in \mathbb{R}^3$ is the mean and $\boldsymbol{\Sigma}_j \in \mathbb{R}^{3\times3}$ is the covariance of the $j-$th distribution. When collecting the real point clouds $\mathcal{P}^{\text{real}}$ we must know if they also include the petiole, when the petiole is present we set $J = 2$, otherwise we set $J = 1$. We need two modes when the petiole is present because we expect one Gaussian to capture the petiole and one to capture the leaf surface. We now call $\mathcal{P}$ the point cloud obtained by adding the points $\mathbf{P}_{\text{sampled}}$ to those present in the skeleton point cloud $\mathcal{S}$. We show the resulting point cloud $\mathcal{P}$ in Fig. 3 for a sugar beet leaf, where one Gaussian is fitted to the petiole and one to the leaf blade.

The output of our 3D U-Net is an offset vector $\mathbf{o} \in \mathbb{R}^3$ for each point in the input point cloud $\mathcal{P}$. We compute the positions of each $u-$ th point $\hat{\mathbf{p}}_u$ in the output point cloud $\hat{\mathcal{P}}$ as $\hat{\mathbf{p}}_u = \mathbf{p}_u + \mathbf{o}_u$.

4 .3 Loss Functions

The objective of our generative function g in Eq. (3), is to generate leaf point clouds $\hat{\mathcal{P}}$ respecting the traits $\mathbf{t}$ defined by the skeletons $\mathcal{S}$, and whose points distribution is close to the real-world one. To achieve this, we combine different loss functions in the training procedure. We divide the loss functions into two main groups. The first group consists of reconstruction loss functions defined on the real-world point cloud $\mathcal{P}_i^{\text{real}}$ from which we extract the skeleton $\mathcal{S}_i$ and the output point cloud $\hat{\mathcal{P}}_i$. The second group consists of loss functions based on the points distribution for all $\mathcal{P}_i^{\text{real}} \in \mathcal{D}_{\text{real}}$. The first group of loss functions aims to produce a leaf point cloud $\hat{\mathcal{P}}$, which respects its skeleton $\mathcal{S}$, looks like the original point cloud $\mathcal{P}^{\text{real}}$ from which $\mathcal{S}$ was extracted, and has a smooth surface. The

second group of loss functions forces the output point cloud $\hat{\mathcal{P}}$ to have a similar point distribution with respect to the point distribution in the real leaf point clouds $\mathcal{P}^{\text{real}}$. Our approach minimizes the total loss

$$\mathcal{L} = \lambda_1 \mathcal{L}_{\text{skeleton}} + \lambda_2 \mathcal{L}_{\text{chamfer}} + \lambda_3 \mathcal{L}_{\text{edges}} + \lambda_4 \mathcal{L}_{\text{smooth}} + \lambda_5 \left(\mathcal{L}_{\text{CMMD}} + \mathcal{L}_{\text{FID}} + \mathcal{L}_{\text{PR}} \right), \tag{6}$$

where we weight the different loss functions using $\lambda_a, a \in \{1, 2, 3, 4, 5\}$. The reconstruction loss functions are $\mathcal{L}_{\text{skeleton}}$, $\mathcal{L}_{\text{chamfer}}$, $\mathcal{L}_{\text{edges}}$, and $\mathcal{L}_{\text{smooth}}$, while $\mathcal{L}_{\text{CMMD}}$, $\mathcal{L}_{\text{FID}}$, and $\mathcal{L}_{\text{PR}}$ are distribution loss functions.

Reconstruction Loss Functions. The reconstruction loss functions use the generated leaf point cloud $\hat{\mathcal{P}}$ and the real leaf point cloud $\mathcal{P}^{\text{real}}$ from which we extracted the skeleton $\mathcal{S}$. Their main purpose is to let the network learn how to generate a leaf respecting the traits $\mathbf{t}$ defined by $\mathcal{S}$. The first term $\mathcal{L}_{\text{skeleton}}$ forces the network to keep the skeleton points $\mathbf{P}_{\text{skeleton}}$ in their original positions, thus preserving the desired traits $\mathbf{t}$. To keep the skeleton points fixed, we enforce that the offsets predicted for those points have all components equal to zero, resulting in the loss term

$$\mathcal{L}_{\text{skeleton}} = \sum_{i}^{\tilde{N}} \mid \mathbf{o}_i \mid \mathbb{1}[\mathbf{p}_i \in \mathcal{S}], \tag{7}$$

where $\mathbb{1}[\mathbf{p}_i \in \mathcal{S}]$ is an indicator function evaluating to 1 when the points $\mathbf{p}_i$ belongs to $\mathcal{S}$.

The second term $\mathcal{L}_{\text{chamfer}}$ is the Chamfer distance. In the literature, this distance is used to evaluate the distance between two sets of points [26, 38]. We include it in our loss to enforce that the points of the generated point cloud $\hat{\mathcal{P}}$ are as close as possible to the points of the real-world point cloud $\mathcal{P}^{\text{real}}$:

$$\mathcal{L}_{\text{chamfer}} = \sum_{\mathbf{p}^{\text{real}} \in \mathcal{P}^{\text{real}}} \min_{\hat{\mathbf{p}} \in \hat{\mathcal{P}}} \left|\left| \mathbf{p}^{\text{real}} - \hat{\mathbf{p}} \right|\right|_2 + \sum_{\hat{\mathbf{p}} \in \hat{\mathcal{P}}} \min_{\hat{\mathbf{p}} \in \hat{\mathcal{P}}} \left|\left| \mathbf{p}^{\text{real}} - \hat{\mathbf{p}} \right|\right|_2, \tag{8}$$

where $\mathbf{p}^{\text{real}} \in \mathbb{R}^3$ is a point belonging to the real-world leaf point cloud $\mathcal{P}^{\text{real}}$. We compute the Chamfer loss in both directions, i.e., we compute the closest point in $\hat{\mathcal{P}}$ for each $\mathbf{p}^{\text{real}}$ and the closest point in $\mathcal{P}^{\text{real}}$ for each $\hat{\mathbf{p}}$.

The third term $\mathcal{L}_{\text{edges}}$ is a regularization loss to enforce that the distance between neighboring points in $\hat{\mathcal{P}}$ is close to a user-defined value $\bar{l}$. This loss enforces that points are evenly distributed in space, penalizing areas that are too sparse or too dense. We compute a k-NN graph over the output point cloud $\hat{\mathcal{P}}$ defining a maximum distance $d_{\max}$ for two points to be connected, i.e., we define an edge $e_{u,v} \in \mathcal{E}$ of length $l_{u,v} = ||\hat{\mathbf{p}}_u - \hat{\mathbf{p}}_v||_2$ between points $\hat{\mathbf{p}}_u$ and $\hat{\mathbf{p}}_v$ if $\hat{\mathbf{p}}_v \in \text{NN}_k(\hat{\mathbf{p}}_u)$, where $\text{NN}_k(\hat{\mathbf{p}}_u)$ is the set of k neighbors with distance from $\hat{\mathbf{p}}_u$ smaller than $d_{\max}$. We then compute the loss as

$$\mathcal{L}_{\text{edges}} = \frac{1}{N \sum_u |\text{NN}_k(\hat{\mathbf{p}}_u)|} \sum_{u=1}^{N} \sum_{v \in \text{NN}_k(\hat{\mathbf{p}}_u)} \left| l_{u,v} - \bar{l} \right|, \tag{9}$$

where $|\text{NN}_k(\hat{\mathbf{p}}_u)|$ is the cardinality of the nearest neighbors of point $\hat{\mathbf{p}}_u$, and $|l_{u,v} - \bar{l}|$ is the absolute distance of edge $l_{u,v}$ from $\bar{l}$.

The last term $\mathcal{L}_{\text{smooth}}$ is a regularization loss to enforce the generated leaf point cloud $\hat{\mathcal{P}}$ to have a smooth surface. This loss acts like a denoising operation and penalizes single points that are too far from their neighbors and that would lead to sharp changes of the leaf surface. We use the edges computed before via the k-NN graph to compute the Laplacian matrix $\mathbf{L} \in \mathbb{R}^{N\times N}$ as

$$\mathbf{L}_{u,v} = \begin{cases} -1 & \text{if } u = v \\ \frac{1}{|NN_k(\hat{\mathbf{p}}_u)|} & \text{if } \exists \mathbf{e}_{u,v} \in \mathcal{E} \\ 0 & \text{otherwise} \end{cases} \tag{10}$$

Then, we compute the Laplacian smoothing objective as $\mathbf{Q} = \mathbf{L}\hat{\mathbf{P}}$, where $\hat{\mathbf{P}} \in \mathbb{R}^{N\times 3}$ is a matrix where each row is a point $\hat{\mathbf{p}}_u \in \hat{\mathcal{P}}$. We define the loss as

$$\mathcal{L}_{\text{smooth}} = \sum_{u=1}^{N} |\mathbf{Q}_u| \,, \tag{11}$$

where $\mathbf{Q}_u \in \mathbb{R}^3$ is the $u-$th row of $\mathbf{Q}$.

Distribution Loss Functions. The second group of loss functions enforces that the distribution of the points in our generated dataset $\mathcal{D}_{\text{ours}}$ of size D_{ours}, is as close as possible to the points distribution of the real-world dataset $\mathcal{D}_{\text{real}}$ of size D_{real}. We use three commonly used metrics for data generation and phrase them as losses. The first term $\mathcal{L}_{\text{CMMD}}$ is the maximum mean discrepancy of the 3D CLIP embeddings [39] given by

$$\begin{aligned}\mathcal{L}_{\text{CMMD}} = &\frac{1}{D_{\text{ours}}(D_{\text{ours}}-1)} \sum_{i=1}^{D_{\text{ours}}} \sum_{j\neq i}^{D_{\text{ours}}} \langle \mathbf{v}_{r,i}^{\text{CLIP}}, \mathbf{v}_{r,j}^{\text{CLIP}} \rangle + \frac{1}{D_{\text{real}}(D_{\text{real}}-1)} \sum_{i=1}^{D_{\text{real}}} \sum_{j\neq i}^{D_{\text{real}}} \langle \mathbf{v}_{f,i}^{\text{CLIP}}, \mathbf{v}_{f,j}^{\text{CLIP}} \rangle \\ &- \frac{2}{D_{\text{ours}} D_{\text{real}}} \sum_{i=1}^{D_{\text{ours}}} \sum_{j=i}^{D_{\text{real}}} \langle \mathbf{v}_{r,i}^{\text{CLIP}}, \mathbf{v}_{f,j}^{\text{CLIP}} \rangle,\end{aligned} \tag{12}$$

where $\mathbf{v}_{r,i}^{\text{CLIP}}$ and $\mathbf{v}_{f,j}^{\text{CLIP}}$ are the CLIP embeddings of the $i-$th real-world point cloud and of the $j-$th generated point cloud. Jayasumana et al. [40] were the first to propose the use of CLIP embeddings, initially for the evaluation of generated images. Exploiting the work by Hegde et al. [39] who provide 3D CLIP embeddings trained on point cloud-image-caption triplets, we compute the CMMD on point clouds.

The second term $\mathcal{L}_{\text{FID}}$ comes from the Fréchet inception distance (FID). As for the previous term, we first compute embeddings for all point clouds, both the real-world $\mathcal{D}_{\text{real}}$ and the generated ones $\mathcal{D}_{\text{ours}}$. We can use the model by Hegde et al. [39] to obtain CLIP embeddings or any other neural network to extract embedding from the point clouds. Once we have the embeddings $\mathbf{v}_{r,i}$ for all the real-world point clouds and $\mathbf{v}_{f,j}$ for all the generated point clouds, we fit Gaussians $\mathcal{N}(\boldsymbol{\mu}_r, \boldsymbol{\sigma}_r)$ and $\mathcal{N}(\boldsymbol{\mu}_f, \boldsymbol{\sigma}_f)$ to the two embedding distribution. We compute the FID as

$$\mathcal{L}_{\text{FID}} = \|\boldsymbol{\mu}_r - \boldsymbol{\mu}_f\|_2 + \text{tr}\left(\boldsymbol{\Sigma}_r + \boldsymbol{\Sigma}_f - 2\sqrt{\boldsymbol{\Sigma}_r \boldsymbol{\Sigma}_f}\right), \tag{13}$$

where $\mathbf{\Sigma}_r$ and $\mathbf{\Sigma}_f$ are the covariance matrices of two distributions, and $\mathrm{tr}(\cdot)$ is the trace operation over the matrix.

The third term $\mathcal{L}_{\mathrm{PR}}$ comes from the precision and recall metrics. These metrics have been extended for evaluating generative approaches by Kynkäänniemi et al. [41]. We shortly explain how the metrics are computed and how we adapt them to use them as losses. As for the previous distribution loss functions, we need point cloud embeddings. We call Φ_r and Φ_f the sets of features extracted from the real and generated point clouds. For each set, we estimate a manifold in the feature space sampling a set of points and surrounding each with a hypersphere that reaches its $k-$ th nearest neighbor. We then evaluate whether an embedding $\mathbf{v}$ is inside the volume estimated from the set of features Φ as

$$b(\mathbf{v}, \Phi) = \begin{cases} 1, \text{ if } \exists_{\mathbf{v}' \in \Phi} : \|\mathbf{v} - \mathbf{v}'\|_2 < \|\mathbf{v}' - \mathrm{NN}_k(\mathbf{v}', \Phi)\|_2 \\ 0, \text{ otherwise} \end{cases} \tag{14}$$

where $\mathrm{NN}_k(\mathbf{v}', \Phi)$ returns the k-th nearest embedding of $\mathbf{v}'$ from Φ. We now compute the precision Pr and recall R as

$$\mathrm{Pr} = \frac{1}{|\mathbf{\Phi}_f|} \sum_{\mathbf{v}_f \in \mathbf{\Phi}_f} b(\mathbf{v}_f, \mathbf{\Phi}_r) \tag{15}$$

$$\mathrm{R} = \frac{1}{|\mathbf{\Phi}_r|} \sum_{\mathbf{v}_r \in \mathbf{\Phi}_r} b(\mathbf{v}_r, \mathbf{\Phi}_f). \tag{16}$$

In contrast to the previous loss functions, which are distances, we cannot use the precision and recall as they are, since we aim to maximize them. Thus, we define the precision-recall loss as

$$\mathcal{L}_{\mathrm{PR}} = \log_{10}\left(\frac{1}{\mathrm{Pr} + \epsilon}\right) + \log_{10}\left(\frac{1}{\mathrm{R} + \epsilon}\right), \tag{17}$$

where ϵ is a small value to ensure numerical stability. In the original paper [41] the authors noticed that the score is inaccurate when measuring the quality of a generated sample that falls into an area of the manifold where only a few real samples are present. Thus, they introduce the realism score

$$\mathrm{Realism}\,(\mathbf{v}_g, \Phi_r) = \max_{\mathbf{v}_r} \left\{ \frac{||\mathbf{v}_r - \mathrm{NN}_k(\mathbf{v}_r, \Phi_r)||_2}{||\mathbf{v}_g - \mathbf{v}_r||_2} \right\}, \tag{18}$$

which is used to filter out elements in such sparse areas of the manifold. The higher the minimum realism score the more we are pruning our manifold Φ_r, thus yielding accurate and higher scores.

Since the real-world data distribution does not change during training, we can easily pre-compute the target values for all the distribution loss functions $\mathcal{L}_{\mathrm{CMMD}}$, $\mathcal{L}_{\mathrm{FID}}$, and $\mathcal{L}_{\mathrm{PR}}$, i.e., $\mathbf{v}_r$, $\mathcal{N}(\boldsymbol{\mu}_r, \boldsymbol{\sigma}_r)$, and Φ_r.

4 .4 Generating New Leaves

Our generative model g takes as input the desired leaf length and width. However, our network needs as input a point cloud $\mathcal{P}$ computed from a skeleton point cloud $\mathcal{S}$, as explained in Sec. 4.2. Thus,

we need to define how to build a skeleton point cloud $\mathcal{S}$ without extracting it from a real-world leaf. As mentioned in Sec. 4 .1, the skeleton consists of three parts: petiole, main axis, and lateral axis. We construct the skeleton in the 3D Cartesian frame, building the main axis along the x direction and the lateral axis along the y direction. The petiole is a line

$$z(x) = \alpha\, x,\; x \in [x_{\min}, 0]\,, \tag{19}$$

where $\alpha \sim \mathcal{U}\left(\frac{\pi}{6}, \frac{\pi}{3}\right)$ and $x_{\min} \sim \mathcal{U}\left(-1, -0.25\right)$. Since we want a 3D point cloud, all these points still need a y coordinate, which we fix to 0. The petiole can be removed from the generative procedure when it's known that the petiole is not present in the training data $\mathcal{D}_{\text{real}}$. We do not use the petiole for the maize leaves since the petiole is not present in the used real-world point clouds $\mathcal{P}^{\text{real}}$. The central axis is defined as a hyperbolic tangent

$$z(x) = \frac{e^x - e^{-x}}{e^x + e^{-x}},\; x \in [0, 1]\,, \tag{20}$$

where we clamp the hyperbolic tangent between $x = 0$, where the petiole starts, and $x = 1$. All points have $y = 0$. We then scale the axis to different sizes.

We define the point where the central axis intersects the lateral axis as $\mathbf{p}_{\text{cross}} = [x_{\text{cross}}, 0, z_{\text{cross}}]^T$, where $x_{\text{cross}} \sim \mathcal{U}(0.25, 0.75)$ and z_{cross} is given by Eq. 20. We use a parabolic function,

$$z(y) = a\, y^2 + b\, y + c \tag{21}$$

to represent the lateral axis, where all points have $x = x_{\text{cross}}$. To compute the parabola coefficients a, b, and c, we need 3 points. One point is $\mathbf{p}_{\text{cross}}$, and two are the extremes on the right and left. We define them as

$$\begin{aligned} \mathbf{p}_r &= [x_{\text{cross}}, 0.5, z_{\text{cross}} + z_{\text{r}}]^\top \\ \mathbf{p}_l &= [x_{\text{cross}}, -0.5, z_{\text{cross}} + z_{\text{l}}]^\top\,, \end{aligned} \tag{22}$$

where z_{r} and z_{l} are two distinct values sampled from $\mathcal{U}(-0.25, 0.25)$. It is important to note that the width of the leaf projected on the y axis is 1 and we can scale it to different sizes. The final skeleton is the collection of points sampled along the curves in Eq. (19), Eq. (20), and Eq. (21). We then scale this parametric skeleton by multiplying all x coordinates of the points for the length scaling factor s_l, and all the y coordinates for the width scaling factor s_w. The scaling factors are the only user-defined parameters that influence the length and width of the generated leaves. Thanks to the different randomly sampled parameters $\alpha, x_{\min}, x_{\text{cross}}, z_{\text{r}}$, and z_{l}, we obtain a large variety of leaves whose lengths and widths are centered on the user desired dimensions.

We compute the final length and width of the leaf using the formula for the computation of arc lengths. The width of leaf L_{width} is computed as

$$L_{\text{width}} = \int_{-s_w}^{s_w} \sqrt{z'(y)^2 + x'(y)^2}\, dy = \int_{-s_w}^{s_w} z'(y)\, dy = \int_{-s_w}^{s_w} (2ay + b)\; dy, \tag{23}$$

where $x'(y) = 0$ because all points on the lateral axis have $x = x_{\text{cross}}$ and we compute $z'(y)$ deriving equation Eq. 21. The length of the leaf L_{length} is computed as

$$L_{\text{length}} = \int_{\hat{x}_{\min}}^{s_l} \sqrt{z'(x)^2 + y'(x)^2}\, dx = \int_{\hat{x}_{\min}}^{0} \alpha\, dx + \int_{0}^{s_l} \left(1 - \tanh^2(x)\right)\, dx, \tag{24}$$

where $\hat{x}_{\min}$ is the resulting minimum value for x we got multiplying $x_{\min}$ for s_l, $y'(x) = 0$ because all points of the main axis have $y = 0$, and we derived Eq. 19 and Eq. 20 to sum the length of the petiole and the length of the leaf blade. We show examples of the skeletons built with our approach in the supplementary material. One can make the skeletons more complex using different functions, or polynomials of higher grade to represent the axes. However, the results of our generative procedure suggest that our skeletons capture the characteristics of the used crop species.

5 Results and Discussion

The main focus of this work is an approach to generate 3D leaf point clouds of known length and width. Using our data improves the performance of trait estimation approaches and enables a more fine-grained analysis of crop growth and productivity. We present our experiments to show the capabilities of our method and to support our key claims: (i) using our generated leaves $\mathcal{D}_{\text{ours}}$ to tune trait estimation approaches perform better than using other generated leaf point clouds; and (ii) all generated leaves respect the leaf traits we condition on and have a high probability of being sampled from the real-world leaf distribution.

5 .1 Experimental Setup

Datasets and Baselines: We use the BonnBeetClouds3D [42] dataset, computed via photogrammetric reconstruction and bundle adjustement, and Pheno4D [43] dataset, captured with a laser scanning system. Both datasets provide single-leaf point clouds. We evaluate our approach by comparing our generated leaf point clouds $\mathcal{D}_{\text{ours}}$ to the leaves generated by three possible g functions in our problem formulation in Eq. (3). First, a set of leaves generated using the procedural agriculture simulation software Helios [31] exported by means of a simulated LiDAR sensor, from now on called $\mathcal{D}_{\text{H}}$. Second, we apply transformations specific to the agricultural domain from our previous work [44] to the leaves obtained from Helios to obtain a larger variety of leaves, that we call $\mathcal{D}_{\text{HT}}$ where HT stands for "Helios + transforms". Third, we train LiDiff [45] to generate leaves conditioned on the skeletons using diffusion, from now on denoted as $\mathcal{D}_{\text{LiDiff}}$. Lastly, we also use the real-world per-plot ground truth data $\mathcal{D}_{\text{real}}$ to highlight the importance of per-leaf traits to improve the performance of the leaf trait estimation methods.

Training Details and Hyperparameters: We train our network using the Adam optimizer [46] with learning rate 0.001. In our loss, we set the weights of the different components to $\lambda_0 = 1$, $\lambda_1 = 0.1$, $\lambda_2 = 0.1$, $\lambda_3 = 10$, $\lambda_4 = 0.01$. These weights help preserve traits while producing realistic leaf point clouds. We use different scaling factors for the different plant species: $s_l \sim \mathcal{U}(0.02, 0.50)$ and $s_w \sim \mathcal{U}(\frac{s_l}{4}, s_l)$ for the sugar beets, $s_l \sim \mathcal{U}(0.15, 0.90)$ and $s_w \sim \mathcal{U}(\frac{s_l}{10}, \frac{s_l}{5})$ for

the maize and $s_l \sim \mathcal{U}(0.10, 0.50)$ and $s_w \sim \mathcal{U}(\frac{s_l}{2}, s_l)$ for the tomato leaves. We use $J = 2$ for sugar beets and tomato leaves, and $J = 1$ for maize leaves. We plan to make our code publicly available upon acceptance.

Metrics: We evaluate the estimated traits by comparing the mean and the standard deviation estimated by all approaches when trained on the different generated data. Additionally, we compute the Fréchet inception distance (FID) [47], the CLIP Maximum Mean Discrepancy (CMMD) [40], and the F-score computed by the precision (Pr) and recall (R) [41] explained in Sec. 4 .3 to estimate how close the distributions of the generated and real data are. We use the pre-trained networks from Mohammadi et al. [48] and Hedge et al. [39] to extract the embeddings $\mathbf{v}$, both networks provide open-source code and pre-trained models. Embedding-based metrics, as the ones employed in our evaluation, are the standard approach to evaluate generative methods [41, 49–52]. These metrics provide a semantic and perceptually relevant comparison, allowing for distribution-level comparisons that would not be possible for distance metrics based on the raw points' positions. To verify that we are not generating the same leaf when conditioned on one specific skeleton, we compute the mean and standard deviation of two different metric distances between multiple leaves $\hat{\mathcal{P}}$ generated from the same skeleton input $\mathcal{S}$.

5 .2 Trait Estimation

The first experiment evaluates how tuning off-the-shelf trait estimation approaches on $\mathcal{D}_{\text{ours}}$ improves the performance compared to other datasets. We show that tuning on $\mathcal{D}_{\text{ours}}$ provides better estimates in terms of mean and standard deviation without relying on costly manual annotations. We test the fine-tuned approaches on the validation set of BonnBeetClouds3D [42], which only provides mean and standard deviation per sub-areas – patches – of the field.

We use three trait estimation approaches f: (1) the approach by Choudhury et al. [12] fits a polynomial to the skeleton of the leaf and then computes the leaf length via integration; (2) the approach by Huang et al. [13] uses the principal components to define the direction of the length and width of the leaf and then computes the longest shortest geodesic distance along those directions via A* [53]; (3) Coherent point drift [14] uses GMMs to find the best correspondences between two set of points. Coherent point drift needs a source point cloud to deform, i.e., a leaf point cloud template, for which we use the leaf template defined by Marks et al. [15]. As explained in Sec. 4.1, this template mesh already defines the points belonging to the main and lateral axes, allowing us to compute the length and width of the leaf after the deformation carried out by Coherent Point Drift.

For $\mathcal{D}_{\text{H}}$, $\mathcal{D}_{\text{HT}}$, and $\mathcal{D}_{\text{ours}}$, we have per-leaf traits, while $\mathcal{D}_{\text{real}}$ only provides per-patch averages. This introduces a systematic error since all leaves from the same plot will have the same ground truth. For $\mathcal{D}_{\text{LiDiff}}$ [45], we use our skeletons of known traits, without changing the noise generation and training procedure. We point out that, for our skeletons, we also know the ground truth leaf angle. However, since this was not in any dataset ground truth measurements, we were not able to use it for evaluation purposes.

In Fig. 4 (a), we show the results testing the approach by Choudhury et al. [12] on the validation patches of the BonnBeetClouds3D dataset. We see that the second patch is the one where tuning

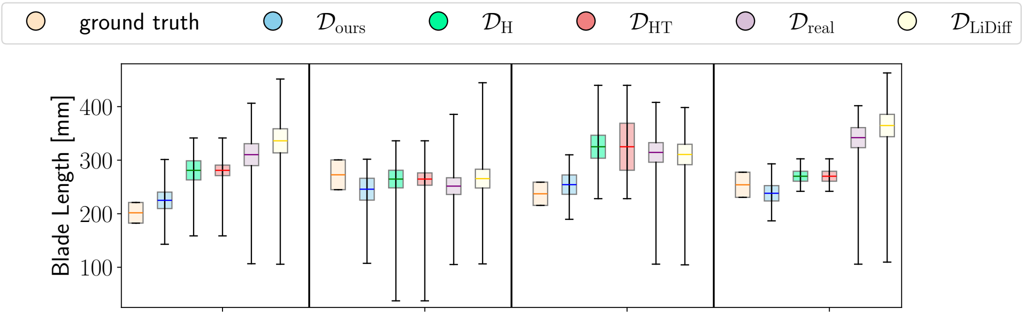

(a) Choudhury et al. [12]

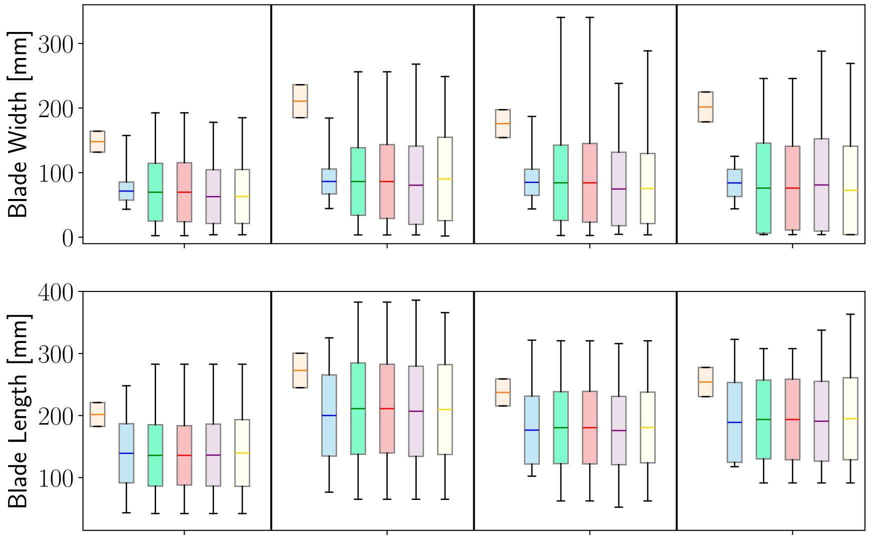

(b) Huang et al. [13]

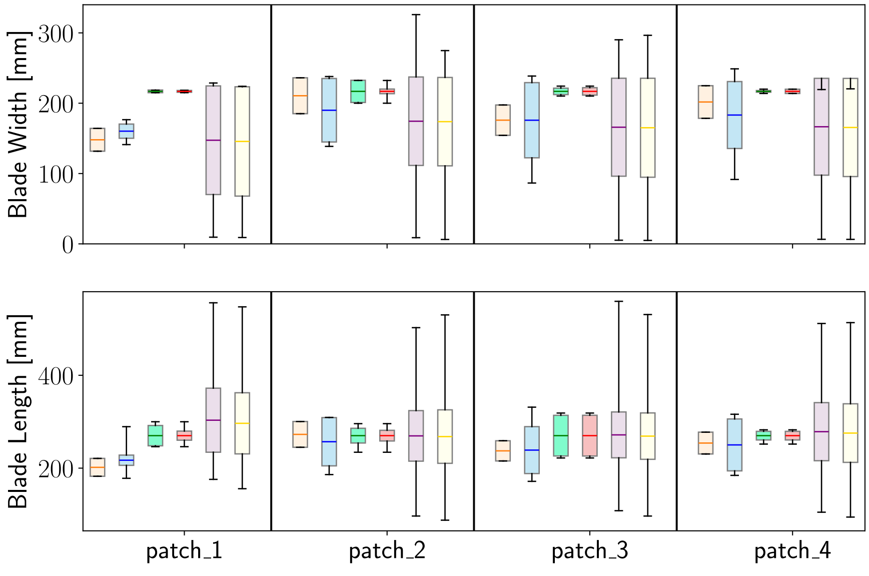

(c) Myronenko et al. [14]

Figure 4: We show the leaf blade length and width estimated by the approaches for BonnBeet-Clouds3D [42] after tuning them on the different datasets. Each bar plot is centered on its mean, the size corresponds to its standard deviation, and we show the maximum and minimum estimated values.

over $\mathcal{D}_{\text{ours}}$ performs worse. This suggests that our generated point clouds do not align well with the leaves in this patch, which is the one with the larger leaves. This could also explain why we are the only one underestimating the size of patch_4, where using $\mathcal{D}_{\text{H}}$ and $\mathcal{D}_{\text{HT}}$ results in smaller variances and better maximum and minimum estimates compared to the other patches. In general, the maximum and minimum estimates obtained tuning on $\mathcal{D}_{\text{ours}}$ are better, even when other datasets provide a mean closer to the ground truth.

The results of Huang et al. [13] shown in Fig. 4 (b) provide similar estimates across all the patches and datasets, suggesting an algorithmic limitation rather than dataset influence. The pipeline has few hyperparameters to remove outliers and define the path cost of the A* algorithm [53] used to compute the leaf blade length and width. We think that the PCA-based method struggles with the complex heart shape of the sugar beet leaf and with occlusions, misidentifying the main axis and, thus, leading to estimate errors. Failures to identify the main axis would also explain the large differences in maximum and minimum estimates. The differences in the results likely depend on the dataset's resolution and sparsity, which would impact the outliers detection and the computation of the distances.

We show in Fig. 4 (c) tuning Coherent point drift [14] on $\mathcal{D}_{\text{ours}}$ provides means closer to the ground truth but with larger standard deviations. The second patch remains problematic, confirming the trend observed for the approach by Choudhury et al. [12]. While $\mathcal{D}_{\text{H}}$ and $\mathcal{D}_{\text{HT}}$ perform better on patch_2, the uniformity of their results suggests a potential overfitting or a failure to capture the data diversity. Tuning the approaches on $\mathcal{D}_{\text{real}}$ yields diverse results but large standard deviations, especially for the blade width, likely because of the lack of per-leaf ground truths. Similarly, also using $\mathcal{D}_{\text{LiDiff}}$ shows high standard deviations, likely because the generation procedure does not preserve the traits accurately. More details about the data generated with LiDiff can be found in the supplementary material.

The results show that using accurate per-leaf traits, even when artificially generated, improves the estimation results on real-world leaves. Our approach enables precise trait estimation without manual labeling or expensive expert knowledge. This is crucial for breeders and agronomists assessing plant traits linked to crop growth and productivity. However, ambiguity in defining leaf width (e.g., midsection vs. widest point) complicates evaluation. Mismatches in width definitions across datasets, generative models, and estimation methods introduce systematic errors, highlighting the need for standardization in trait measurement.

5 .3 Realistic Data Generation

The second set of experiments assesses how closely our generated leaf point clouds match real-world distributions. We demonstrate that our approach generates leaf point clouds with features similar to real-world data, making them valuable for tuning trait estimation approaches to use in real-world scenarios. Additionally, our method generated diverse leaves while maintaining specified blade length and width, bridging the gap between simulated and real-world data. As detailed in Sec. 5 .1, we evaluate the generated points clouds with the metrics explained in Sec. 4 .3. They compare distributions of embeddings, which we extract using the two different pre-trained networks

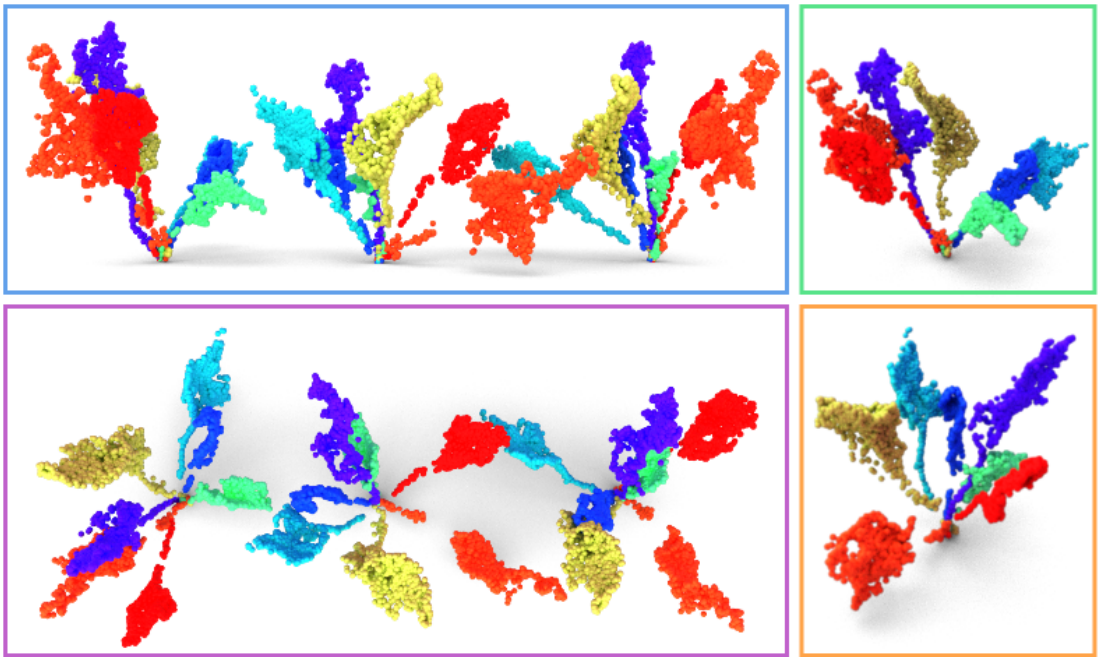

Figure 5: Examples of sugar beet leaves generated by our approach with different leaf angles, stem lengths, and blade lengths and widths. We show a side (blue rectangle) and a top view (purple rectangle) of three plants generated using the same leaves with different orientations and positions. We show zoomed in views of the first (green rectangle) and second plant (orange rectangle).

mentioned in Sec. 5.1.

5 .3.1 Leaf Distribution

We use the validation set from BonnBeetClouds3D, from now on called SugarBeets dataset, and the unlabeled plants from Pheno4D as real-world target point clouds. We show examples of the leaves generated by our approach trained on SugarBeets in Fig. 5, where we use all the information we have thanks to the skeleton to also merge our generated leaves into complete plants. We can see that the network learned different leaf shapes that are not uniquely connected to the leaf dimensions. For example, the yellow and green leaves have a similar shape even if the green leaf is smaller. We can also modify the stem angle, giving the desired orientation to each leaf; this is clear for the orange leaf whose is almost vertical in the left plant and almost horizontal in the central plant. Looking at the light blue leaf, we can see that we are also able to rotate the leaf around the stem axis, thus changing the surface orientation. We compute all metrics for $\mathcal{D}_{\text{ours}}$, $\mathcal{D}_{\text{H}}$, $\mathcal{D}_{\text{HT}}$ and $\mathcal{D}_{\text{LiDiff}}$. Since LiDiff requires conditioning on skeletons but does not provide a skeleton generation procedure, we use the skeletons of the training set to generate new leaves, potentially giving it an advantage over methods relying on domain expertise or procedurally generated skeletons. Tab. 1 shows the results of the CMMD, FID, and F-score. For the F-score, we use both feature extractors, i.e., the network by Mohammadi et al. [48] and by Hedge et al. [39], to isolate network-specific influences. Fitting a Gaussian on the CLIP embeddings of the real-world point clouds was failing and starting

Dataset	Generated Data	FID ↓	F-score ↑	F-score + CLIP ↑	CMMD ↓
SugarBeets	$\mathcal{D}_{\text{H}}$	312.84	0.01	0.01	169.71
	$\mathcal{D}_{\text{HT}}$	14.01	**0.36**	0.01	28.45
	$\mathcal{D}_{\text{LiDiff}}$	26.89	0.35	0.11	22.05
	$\mathcal{D}_{\text{ours,rec}}$	108.73	0.03	0.17	20.46
	$\mathcal{D}_{\text{ours}}$	**13.71**	0.21	**0.20**	**19.53**
Maize	$\mathcal{D}_{\text{H}}$	11.28	0.19	0.02	29.39
	$\mathcal{D}_{\text{HT}}$	0.17	0.39	0.17	16.36
	$\mathcal{D}_{\text{LiDiff}}$	1.05	0.22	0.09	65.14
	$\mathcal{D}_{\text{ours}}$	**0.12**	**0.55**	**0.36**	**11.51**
Tomato	$\mathcal{D}_{\text{H}}$	7.89	0.01	0.06	28.26
	$\mathcal{D}_{\text{HT}}$	5.29	0.02	0.06	24.16
	$\mathcal{D}_{\text{LiDiff}}$	6.66	0.05	0.10	65.42
	$\mathcal{D}_{\text{ours}}$	**2.76**	**0.15**	**0.17**	**12.39**

Table 1: Evaluation of the Fréchet inception distance (FID), F-score, and CLIP Maximum Mean Discrepancy (CMMD) for the leaf point clouds generated by the different approaches compared to the test sets. Our approach outperforms the others on most metrics across the different datasets. Our results are highlighted using gray colored rows.

with non-default initializations provided inconsistent results, thus we do not include the FID metric with CLIP embeddings. Since the improved precision and recall metrics depend on the number of neighbors used to construct the real and generated data manifolds (Φ_r and Φ_f), we evaluate the F-score across multiple values of k, specifically $k \in \{2, 4, 8, 16, 32, 48, 64, 96\}$. We then report the mean F-score over these values to provide a more stable and robust estimate.

We see that applying our domain-specific transforms to $\mathcal{D}_{\text{H}}$ improves all metrics across all datasets. The results are generally better on Pheno4D, likely because they provide leaves at different growth stages while the SugarBeets dataset was recorded over the same day. Overfitting to the exact growth stage could lead to a boost, explaining the results of LiDiff which uses the skeleton of the training point clouds. Our approach outperforms the others across most of the investigated scenarios, except for FID + CLIP and the F-score on the SugarBeets dataset, where the feature extractors yield conflicting results. For the SugarBeets dataset we also provide an ablation study on our approach, namely $\mathcal{D}_{\text{ours,rec}}$, where we keep everything the same but we train using only the reconstruction losses, without the distribution ones. We can see that using the distribution losses improves all metrics, especially the FID and the F-score that get 7 times better. This shows the contribution of our distribution loss functions and the importance of a distribution supervision while generating the leaf point clouds.

Given the low F-score values in Tab. 1, we compute the realism score as in Eq. 18 and re-evaluate the generative approaches on the SugarBeets dataset, where the two feature extractors contradict each other. We noticed that the number of samples filtered out by the realism score was high, especially for low values of k. Tab. 2 shows the F-score filtering out elements with low realism and considering only results where more than half of the generated leaves were used. Most results improve while increasing the minimum realism score, but many approaches fail when the minimum

Realism	Generated Data	F-score ↑	F-score + CLIP ↑
0.0	$\mathcal{D}_{\mathrm{H}}$	0.01	0.01
	$\mathcal{D}_{\mathrm{HT}}$	**0.36**	0.01
	$\mathcal{D}_{\mathrm{LiDiff}}$	0.35	0.11
	$\mathcal{D}_{\mathrm{ours}}$	0.21	**0.20**
0.5	$\mathcal{D}_{\mathrm{H}}$	0.01	0.01
	$\mathcal{D}_{\mathrm{HT}}$	**0.48**	0.02
	$\mathcal{D}_{\mathrm{LiDiff}}$	0.33	0.11
	$\mathcal{D}_{\mathrm{ours}}$	0.22	**0.23**
1.0	$\mathcal{D}_{\mathrm{H}}$	0.02	0.02
	$\mathcal{D}_{\mathrm{HT}}$	0.45	0.04
	$\mathcal{D}_{\mathrm{LiDiff}}$	**0.64**	0.11
	$\mathcal{D}_{\mathrm{ours}}$	0.29	**0.55**
1.5	$\mathcal{D}_{\mathrm{H}}$	-	-
	$\mathcal{D}_{\mathrm{HT}}$	**0.80**	-
	$\mathcal{D}_{\mathrm{LiDiff}}$	-	-
	$\mathcal{D}_{\mathrm{ours}}$	0.29	**0.90**

Table 2: F-score computed for all the approaches using different values of realism to filter out the outliers. When less than half of the samples were valid, we do not report any result (-). The more samples we filter out, the higher the metrics. Our approach is the only one that always provides enough samples in the dense area of the distribution.

accepted realism is too high. When realism exceeds 1.0, only our generated leaves consistently allow F-score computation with both models, indicating strong alignment with real-world distributions. This explains why tuning on our data enhances leaf trait estimation. However, the feature extractors still disagree, highlighting the need for a standardized feature extractor, as it exists in the image domain, or even a domain-specific feature extractor that better captures important features in the agricultural domain.

5 .3.2 Leaf Variety

Our method generates leaf point clouds from skeletons, but we want to ensure diversity by generating different leaves given the same skeleton. This enhances the dataset variety without altering skeleton-building procedures or training multiple generative networks g. A diverse dataset is crucial to optimize trait estimation approaches avoiding overfitting to common samples. In this experiment, we input the same skeleton multiple times and compare the generated leaves by computing two distances. First, we use the Chamfer distance. Second, we compute the meshes from the leaf point clouds via ball pivoting [54] and measure the surface differences as the differences of the distances from the meshes to randomly sampled 3D points. In Fig. 6, we show a simplified 2D example of the point-to-mesh distance.

In Tab. 3, we report the mean chamfer and point-to-mesh distances on 10 leaves generated with the same skeleton averaged over 10 runs. Since we cannot condition the Helios software on a skeleton, we report the results only for our approach and LiDiff. While the chamfer distances have similar

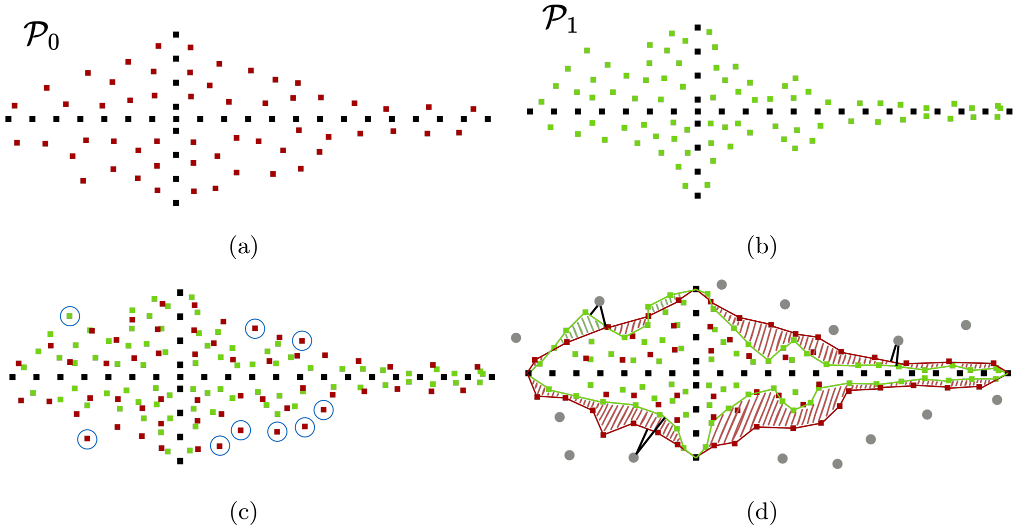

Figure 6: (a) and (b) are two leaves generated from the same skeleton. We show in (c) that when using the Chamfer distance, the outliers in the blue circles are the only ones providing a meaningful distance since most of the points are in the same area. In (d), we show our proposed point-to-mesh distance, where we compute the difference between the distances from each gray point to the two meshes.

Generated Data	Chamfer [mm]		point-to-mesh [mm]	
	mean	std	mean	std
$\mathcal{D}_{\text{LiDiff}}$	6.14	7.53	55.82	57.87
$\mathcal{D}_{\text{ours}}$	5.69	1.69	67.69	17.58

Table 3: Average mean and standard deviation for the chamfer and point-to-mesh distances, computed over 10 trials on 10 leaves generated conditioning the network with the same skeleton.

means, LiDiff's standard deviation is approx. 4.5 times higher, likely due to the weaker compliance to the skeleton. For the point-to-mesh distance, we see a larger difference in the mean, more than 1 cm, and again the standard deviation of LiDiff is more than 3 times ours.

Given our previously reported results, we think that our approach provides more precise per-leaf ground truths for tuning trait estimation methods. In contrast, LiDiff produces a wider variety of leaves, at the cost of higher leaf trait errors. Since LiDiff is a general-purpose approach for conditioned diffusion, adding specific losses could help the approach follow more closely the input skeleton and improve the results.

6 Limitations and Future Work

In this article, we tested our approach on different crop varieties, all exhibiting similar shape complexity. Our approach works also on more complex shapes, as compound leaves – leaves where the blade is divided into two or more leaflets, if enough data is provided. Since the network learns from

real-world data, we can use the whole compound leaf as it is for the network to learn its shape. Nonetheless, structural complexity presents additional challenges, and some enhancements could improve the convergence speed and the overall performance. One improvement involves modifying the algorithm for building the skeleton. For example, we could combine multiple skeletons to capture the morphology of a compound leaf. Additionally, adjusting the number of Gaussians J in the GMM can improve the initial position of the points, leading to more efficient training. While these changes are not strictly required, they could reduce the need for training data and provide a better a more effective initialization. Another potential direction is to learn the shape of single leaflets instead of the entire compound leaf. This method wouldn't require algorithmic changes, but it would require access to single leaflet point clouds – harder to obtain than single leaves. Furthermore, knowledge about the leaf structure would be needed to reconstruct complete leaves from the generated leaflets.

As with any deep-learning method, our approach assumes that the distributions of the training and inference data is similar. When there is a mismatch – such as training on simple low-resolution skeletons and then performing inference on complex and high-resolution skeletons, or vice versa – the performance may degrade. However, this is the only assumption we make on the skeletons. As demonstrated in our experiments, our methods works with different skeletons extracted by different methods without requiring adaptation.

For future work, we aim to evaluate our approach on more fine-grained tasks, such as generating distinct varieties within a single crop species. This would require large and variety-specific datasets, as the network must learn finer details. Currently, the primary limitation for this direction is the lack of available data. Moreover, existing evaluation metrics often rely on networks pre-trained on large datasets of common objects [55, 56], which may struggle to differentiate between single crop species varieties. A domain-specific foundation model tailored to plant data would yield more reliable evaluations. Another possible direction for future research is integrating our method with plant growth models. Since our network can be trained on leaves from specific growth stages, we can generate a large variety of leaves for each stage. Plant growth models could provide the expected leaf blade length and width based on environmental and nutritional input, allowing for more realistic simulations without the need for external user-defined inputs.

7 Conclusions

In this paper, we address the data bottleneck when working with 3D point clouds of leaves and presented an approach that can generate realistic leaf point clouds with given traits, such as leaf length and width. Our method generates realistic leaves that can then be directly used to train or fine-tune off-the-shelf leaf trait estimation approaches. We have shown that optimizing the hyperparameters of different methods on our generated data achieves better results than using per-plot ground truth averages or leaves generated by other state-of-the-art approaches. Using our data allows for more precise and fine-grained estimation of traits that directly influence crop growth and productivity. Our results suggest that per-leaf ground truth data is essential for estimating leaf traits and that generated data can significantly boost the performance of existing methods. We run experiments on three different plant species, demonstrating the potential of learning from real-world data with-

out requiring labels. Even when real-world per-leaf ground truth measurements are available, our approach can generate leaves of different lengths and widths to fill potential gaps in the collected data. Thus, we can obtain unbiased datasets from different growth stages, reducing the efforts of the experts and the need for destructive measurements.

Author Contributions

The authors confirm contribution to the paper as follow: study conception and design: G. Roggiolani; interpretation of results: G. Roggiolani; draft manuscript: G. Roggiolani, Brian. N. Bailey, J. Behley, C. Stachniss; funding acquisition and project coordination: C. Stachniss. All authors reviewed the results and approved the final version of the manuscript.

Funding

This work has partially been funded by the Deutsche Forschungsgemeinschaft (DFG, German Research Foundation) under Germany's Excellence Strategy, EXC-2070 – 390732324 – PhenoRob.

Conflicts of Interest

The authors declare that there is no conflict of interest regarding the publication of this article.

Data Availability

We use two publicly available datasets. Pheno4D [43] is available at the url: `https://www.ipb.uni-bonn.de/data/pheno4d/index.html`. It contains maize and tomato plants measured daily,over 12 and 20 days respectively. Only half of the point cloud are labeled with temporally consistent labels. The SugarBeets dataset BonnBeetClouds3D [42], available at `https://bonnbeetclouds3d.ipb.uni-bonn.de/`, and it contains point clouds from a 1.5 m by 7 m field plot. The training set consists of 128 plants, with a total of 1782 leaves; the validation set consists of 17 plants, with a total of 260 leaves. The dataset provides labels for single plants and leaves, and the keypoints of the leaf base, tip, right and left corner, for each labeled leaf. The leaf blade length and width, together with the petiole length and width, are given per plot, where a plot is a 1 m by 1 m patch of the field.

Supplementary Materials

S .1 Problem Decomposition

The main problem we aim to solve with our approach is the lack of accurate leaf trait annotations needed to optimize trait estimation methods. Our solution is to generate synthetic leaves, for which we know the accurate traits. Such generated data can be used to tune the trait estimation approaches, which can then be deployed on real-world leaves, obtaining more accurate results. We provide an overview of how we decompose the problem of generating the data for traits estimation approaches in Fig. S.1. Firstly, we separate the generation from the trait estimation, which are

depicted in the upper and lower purple blocks. Each purple block has a blue block inside, which corresponds to the pure inference pipeline that is run after the block has already been optimized.

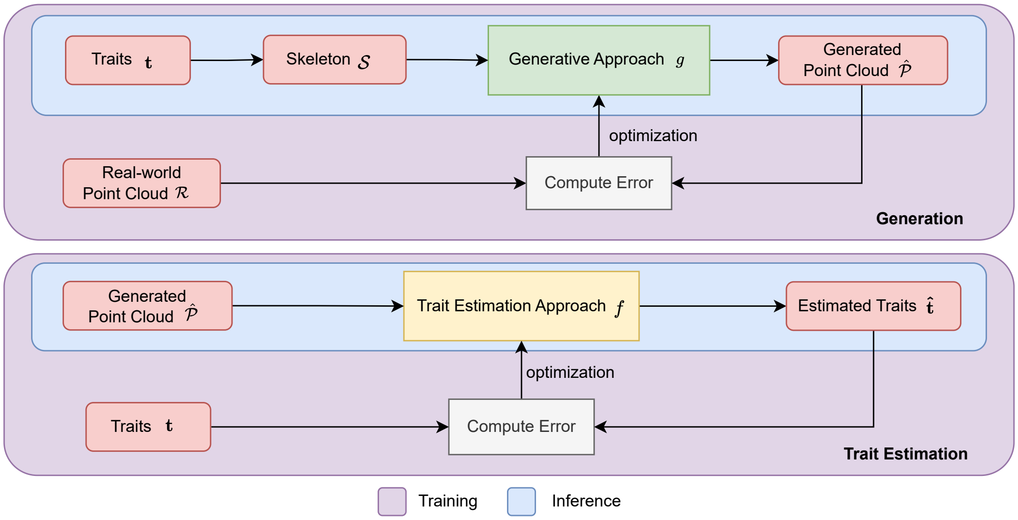

Figure S.1: Outline of the training and inference procedure for generating a leaf point clouds $\hat{\mathcal{P}}_i$, with given traits $\mathbf{t}_i$ (top), and for estimating leaf traits $\hat{\mathbf{t}}_i$ for a given point cloud $\hat{\mathcal{P}}_i$ (bottom). We highlight the generative approach g in green and the trait estimation approach f in yellow. The training procedure is colored in purple, while the inference is in blue.

When we focus on the upper block, i.e., the block about the generative method, we see that at inference time our input is a vector $\mathbf{t}$ of desired traits. We then build a skeleton point cloud $\mathcal{S}$ reflecting the desired input traits. In our article, we explain how we augment the skeleton point cloud to obtain the input for our network. However, considering the augmentation as part of our generative approach, we can use the point cloud skeleton $\mathcal{S}$ as the input of the generative approach g, depicted in green. The output of the generative approach is a point cloud $\hat{\mathcal{P}}$. To obtain as output a realistic leaf point cloud that respects the input traits, the generative approach must be optimized. Outside the blue block, we can optimize the generative approach by minimizing the error between the generated leaf point cloud and the real-world point clouds. We explain each component of our loss function in Sec. 4.3 of the article.

In the lower block, i.e., the block about the trait estimation method, we see that the trait estimation approach f, depicted in yellow, only takes as input a generated point cloud $\hat{\mathcal{P}}$. In the article, we cover how the different off-the-shelf estimation approaches use the raw point cloud to estimate the leaf blade length and width. The output of the approach f is, thus, a set of traits $\hat{\mathbf{t}}$ estimated for the current point cloud in input. As for the generative method, to obtain a good trait estimation, the parameters of the estimation approach f must be optimized. Since the output is the set of traits $\hat{\mathbf{t}}$, we need to compute the error between the estimated traits and the real traits $\mathbf{t}$ associated with the input point cloud. To optimize the trait estimation methods, we compute the error as the absolute distance between the estimated and ground truth trait.

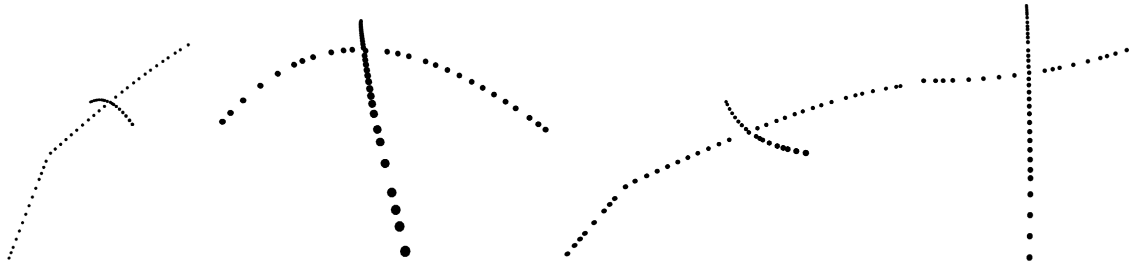

(a) (b) (c) (d)

Figure S.2: Generated skeletons seen from the side [(a) and (c)] and from the petiole [(b) and (d)]. The axis angle, the petiole length, and the extremities of the lateral axis vary.

S .2 Skeletons Examples

One crucial part of our approach is the ability to produce different types of skeletons to guide the generative approach into creating a large variety of realistic leaves. We do not only consider the two traits we use for the evaluation, i.e., the leaf blade length and width, but also other characteristics to increase the differences between our generated leaves. We vary the length of the stem, the leaf angle between the stem and the blade, the position at which the main and lateral axes intersect, and the orientation and the skewness of the parabola representing the lateral axis.

We show two exemplary skeletons in Fig. S.2. We can see that the skeletons have different stem lengths, leaf angles, and intersection points of the two axes; this is mostly clear from the side view of the skeletons [(a) and (c)]. The two skeletons also present a lateral axis with opposite orientation. In the views from the petiole [(b) and (d)], we can see that the lateral axes are skewed, not being perfectly symmetrical with respect to the main axis.

We point out that our approach generates different leaves also when starting from the exact same skeleton, as explained in Sec. 5.3.2. This ulteriorly increases the number of possible leaves we generate, allowing our generated data to cover more and more of the distribution of the real-world leaves' shapes and sizes.

S .3 Failure Cases of Diffusion

The main point of our approach is to generate realistic point clouds of known leaf length and width. The realistic part is important to avoid a wide gap between the dataset and the real-world leaves, on which we want to apply the trait estimation approaches. Knowing the accurate leaf length and width is essential to optimize the trait estimation approaches and obtain parameters that work well on real-world leaves.

Here, we provide some qualitative examples of the dataset generated by LiDiff (diffusion-based baseline) and explain the main drawback of using this dataset to tune the trait estimation methods. We show in Fig. S.3 two exemplary leaves generated by LiDiff. We see that the leaves look realistic, qualitatively resembling leaves. However, the leaves generated by LiDiff do not accurately follow the skeletons, depicted as black dots, representing our desired input traits. In Fig. S.3 (a), the leaf is shorter than expected, leaving one skeleton point outside of the leaf blade, thus providing an

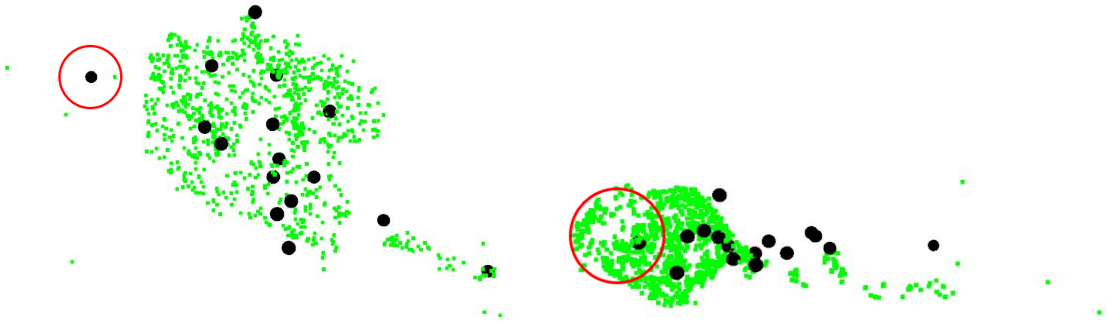

(a) (b)

Figure S.3: Two leaves generated by LiDiff, where we show in black dots the skeleton used for the conditioning and in green the generated point clouds. The point clouds have the appearance of leaves, but do not respect the skeleton traits. We point out the errors in red circles.

incorrect leaf length. In Fig. S.3 (b), we see the opposite problem; the last skeleton point is actually inside the leaf blade that overshoots the expected leaf length.

This problem is hard to address because it is not systematic, i.e., we do not always have shorter or always longer leaves. In that case, we could estimate the systematic error and use it to correct the ground truth measures associated with the generated leaves. However, since this is not the case, we are left with inaccurate leaf traits. Using inaccurate traits during the optimization leads to the same problem of using the ground truth "per plot" measures. This is why, in Sec. 5.2 of the article, the results of the trait estimation approaches tuned on the data generated by LiDiff have large standard deviations and larger errors in the means of the estimated traits.

S .4 Dataset Images

To better understand the differences between the leaves of the different crop species we used in the article, we include images from all three crop species. We show in Fig. S.4 images of sugar beets plants from BonnBeetClouds3D [42] [(a) – (f)], tomato plants from Pheno4D [43] [(i), (j), and (n) –(p)], and maize plants from Pheno4D [43] [(g), (h), and (k)–(m)]. We used the labels of the leaf instances to assign a different color to each leaf for all the plants. For the tomato and maize plants, we show one plant from the first date [(g)–(j)] and the same plant from the last date [(k)–(p)]. We can see that leaves at the early stages are more similar, but they grow into very different shapes and structures in the later growth stages. For the sugar beets, we do not have access to earlier growth stages, so we present two distinct plants from the dataset. However, sugar beets are dicotyledonous plants, thus resembling tomato plants in the early growth stages [(g) and (h)]. Since BonnBeetClouds3D [42] was captured in the field and not in a controlled environment, we can see that the bottom part of the plant is occluded and not entirely present in the point cloud, which increases the challenge in estimating the correct traits.

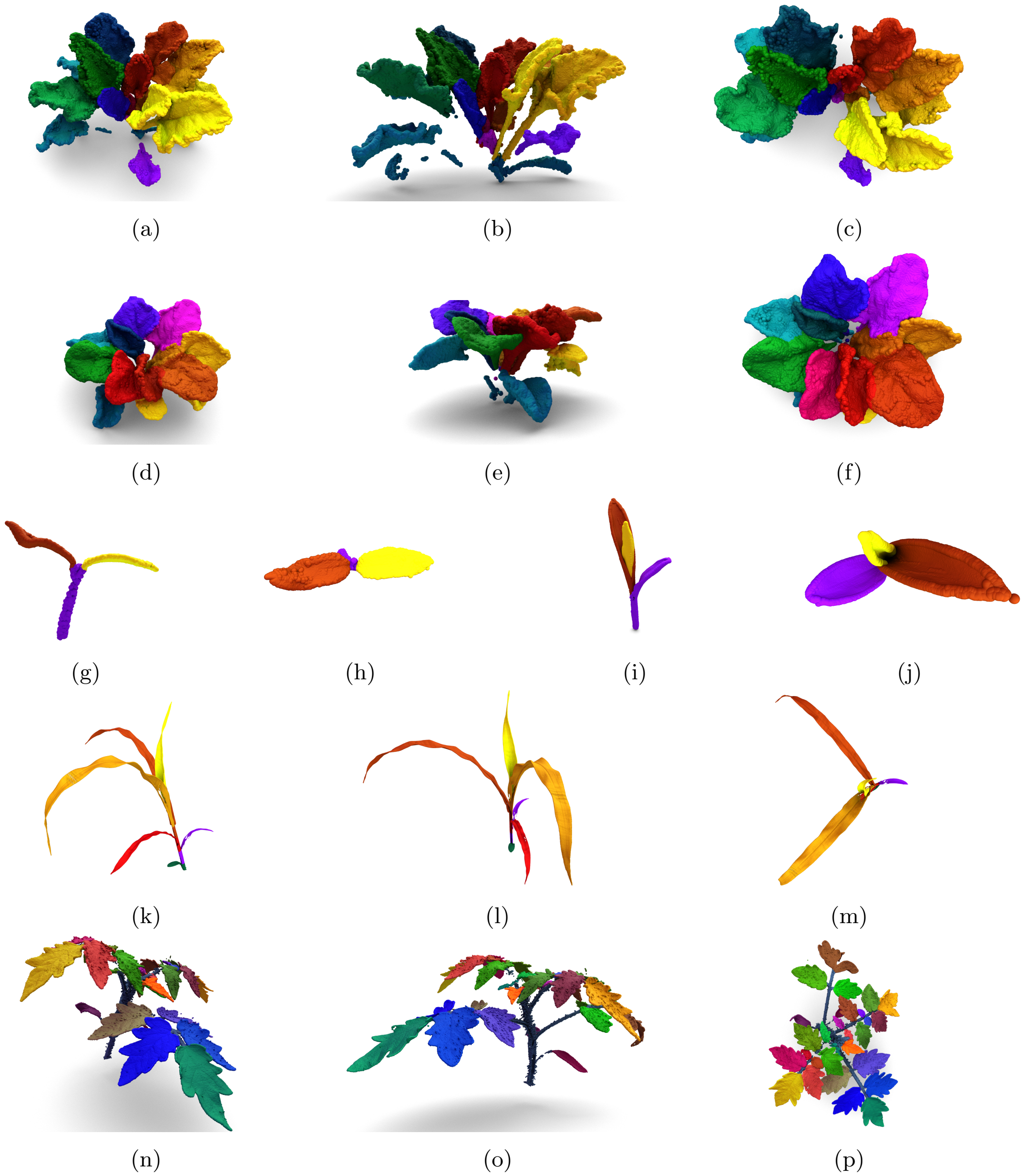

Figure S.4: Exemplary plants from the three datasets used in the article. (a) – (f) are from BonnBeetClouds3D [42], (g), (h), and (k)–(m) are maize plants, while (i), (j), and (n) –(p) are tomato plants from Pheno4D [43]. For Pheno4D we show the same plants at the first date (g)–(j) and at the last date (k)–(p) of data acquisition. For every plant, one different color is assigned to a unique leaf.

References

1. Tabe-Ojong MPJ, Lokossou JC, Gebrekidan B, and Affognon HD. Adoption of climate-resilient groundnut varieties increases agricultural production, consumption, and smallholder commercialization in West Africa. Nature Communications 2023;14:5175.

2. Chen S, Guo Y, Sirault X, Stefanova K, Saradadevi R, Turner NC, Nelson MN, Furbank RT, Siddique KH, and Cowling WA. Nondestructive phenomic tools for the prediction of heat and drought tolerance at anthesis in Brassica species. Plant Phenomics 2019.
3. Leonetti P, Hanafy MS, Tayade R, Ramakrishnan M, Sonah H, and Jacobsen HJ. Leveraging genomics, phenomics, and plant biotechnology approaches for improving abiotic and biotic stress tolerance in cereals and legumes. Frontiers in Plant Science 2023;14:1307390.
4. Migicovsky Z, Li M, Chitwood DH, and Myles S. Morphometrics Reveals Complex and Heritable Apple Leaf Shapes. Frontiers in Plant Science 2018;8.
5. Tamang BG, Zhang Y, Zambrano MA, and Ainsworth EA. Anatomical determinants of gas exchange and hydraulics vary with leaf shape in soybean. Annals of Botany 2022;131:909–20.
6. Singh B, Kumar S, Elangovan A, Vasht D, Arya S, Duc NT, Swami P, Pawar GS, Raju D, Krishna H, et al. Phenomics based prediction of plant biomass and leaf area in wheat using machine learning approaches. Frontiers in Plant Science 2023;14:1214801.
7. Yang W, Feng H, Zhang X, Zhang J, Doonan JH, Batchelor WD, Xiong L, and Yan J. Crop phenomics and high-throughput phenotyping: past decades, current challenges, and future perspectives. Molecular Plant 2020;13:187–214.
8. Mahmood R, Lucas J, Acuna D, Li D, Philion J, Alvarez JM, Yu Z, Fidler S, and Law MT. How Much More Data Do I Need? Estimating Requirements for Downstream Tasks. In: 2022:275–84.
9. Ubbens J, Cieslak M, Prusinkiewicz P, and Stavness I. The use of plant models in deep learning: an application to leaf counting in rosette plants. Plant Methods 2018;14:1–10.
10. Weyler J, Milioto A, Falck T, Behley J, and Stachniss C. Joint Plant Instance Detection and Leaf Count Estimation for In-Field Plant Phenotyping. IEEE Robotics and Automation Letters (RA-L) 2021;6:3599–606.
11. Luo L, Jiang X, Yang Y, Samy ERA, Lefsrud M, Hoyos-Villegas V, and Sun S. Eff-3DPSeg: 3D Organ-Level Plant Shoot Segmentation Using Annotation-Efficient Deep Learning. Plant Phenomics 2023;5:0080.
12. Das Choudhury S, Maturu S, Samal A, Stoerger V, and Awada T. Leveraging image analysis to compute 3D plant phenotypes based on voxel-grid plant reconstruction. Frontiers in Plant Science 2020;11:521431.
13. Huang X, Zheng S, and Gui L. Automatic measurement of morphological traits of typical leaf samples. Sensors 2021;21:2247.
14. Myronenko A and Song X. Point set registration: Coherent point drift. IEEE Trans. on Pattern Analysis and Machine Intelligence (TPAMI) 2010;32:2262–75.
15. Marks E, Magistri F, and Stachniss C. Precise 3D Reconstruction of Plants from UAV Imagery Combining Bundle Adjustment and Template Matching. In: *Proc. of the IEEE Intl. Conf. on Robotics & Automation (ICRA).* 2022.

16. Marks E, Sodano M, Magistri F, Wiesmann L, Desai D, Marcuzzi R, Behley J, and Stachniss C. High Precision Leaf Instance Segmentation for Phenotyping in Point Clouds Obtained Under Real Field Conditions. IEEE Robotics and Automation Letters (RA-L) 2023;8:4791–8.

17. Chen F, Tsaftaris SA, and Giuffrida MV. GMT: Guided Mask Transformer for Leaf Instance Segmentation. 2024;arXiv:2406.17109.

18. Guo W, Carroll ME, Singh A, Swetnam TL, Merchant N, Sarkar S, Singh AK, and Ganapathysubramanian B. UAS-Based Plant Phenotyping for Research and Breeding Applications. Plant Phenomics 2021;2021.

19. Esser F, Rosu RA, Cornelißen A, Klingbeil L, Kuhlmann H, and Behnke S. Field Robot for High-Throughput and High-Resolution 3D Plant Phenotyping: Towards Efficient and Sustainable Crop Production. IEEE Robotics and Automation Magazine (RAM) 2023;30:20–9.

20. Kolhar S and Jagtap J. Plant trait estimation and classification studies in plant phenotyping using machine vision–A review. Information Processing in Agriculture 2023;10:114–35.

21. Pape JM and Klukas C. 3-D Histogram-Based Segmentation and Leaf Detection for Rosette Plants. In: *Proc. of the Europ. Conf. on Computer Vision Workshops*. 2015:61–74.

22. Bai G, Ge Y, Hussain W, Baenziger PS, and Graef G. A multi-sensor system for high throughput field phenotyping in soybean and wheat breeding. Computers and Electronics in Agriculture 2016;128:181–92.

23. Wang Z, Wang K, Yang F, Pan S, and Han H. Image Segmentation of Overlapping Leaves Based on Chan–Vese Model and Sobel Operator. Information Processing in Agriculture 2017;5:1–10.

24. Roggiolani G, Sodano M, Guadagnino T, Magistri F, Behley J, and Stachniss C. Hierarchical Approach for Joint Semantic, Plant Instance, and Leaf Instance Segmentation in the Agricultural Domain. In: *Proc. of the IEEE Intl. Conf. on Robotics & Automation (ICRA)*. 2023.

25. Ge Y, Bai G, Stoerger V, and Schnable JC. Temporal dynamics of maize plant growth, water use, and leaf water content using automated high throughput RGB and hyperspectral imaging. Computers and Electronics in Agriculture 2016;127:625–32.

26. Magistri F, Marcuzzi R, Marks E, Sodano M, Behley J, and Stachniss C. Efficient and Accurate Transformer-Based 3D Shape Completion and Reconstruction of Fruits for Agricultural Robots. In: *Proc. of the IEEE Intl. Conf. on Robotics & Automation (ICRA)*. 2024.

27. Xue W, Ding H, Jin T, Meng J, Wang S, Liu Z, Ma X, and Li J. CucumberAI: Cucumber Fruit Morphology Identification System Based on Artificial Intelligence. Plant Phenomics 2024;6:0193.

28. Carbone C, Potena C, and Nardi D. Simulation of near Infrared Sensor in Unity for Plant-weed Segmentation Classification. In: *Proc. of the Intl. Conf. on Simulation and Modeling Methodologies, Technologies and Applications*. 2020.

29. Li T, Burridge J, Blok PM, and Guo W. A Patch-Level Data Synthesis Pipeline Enhances Species-Level Crop and Weed Segmentation in Natural Agricultural Scenes. Agriculture 2025;15.

30. Helmrich DN, Bauer FM, Giraud M, Schnepf A, Göbbert JH, Scharr H, Hvannberg ET, and Riedel M. A scalable pipeline to create synthetic datasets from functional–structural plant models for deep learning. in silico Plants 2024;6:diad022.

31. Bailey BN. Helios: A Scalable 3D Plant and Environmental Biophysical Modeling Framework. Frontiers in Plant Science 2019;10:1185.

32. Choi T, Guevara D, Cheng Z, Bandodkar G, Wang C, Bailey BN, Earles M, and Liu X. DAVIS-Ag: A Synthetic Plant Dataset for Prototyping Domain-Inspired Active Vision in Agricultural Robots. In: *Proc. of the Intl. Conf. on Automation Science and Engineering (CASE)*. 2024.

33. Triggs B, McLauchlan PF, Hartley RI, and Fitzgibbon AW. Bundle Adjustment - A Modern Synthesis. In: *Proc. of the Intl. Workshop on Vision Algorithms: Theory and Practice*. 1999.

34. Magistri F, Chebrolu N, and Stachniss C. Segmentation-Based 4D Registration of Plants Point Clouds for Phenotyping. In: *Proc. of the IEEE/RSJ Intl. Conf. on Intelligent Robots and Systems (IROS)*. 2020.

35. Ronneberger O, P.Fischer, and Brox T. U-Net: Convolutional Networks for Biomedical Image Segmentation. In: *Proc. of the Medical Image Computing and Computer-Assisted Intervention (MICCAI)*. 2015.

36. Thomas H, Qi C, Deschaud J, Marcotegui B, Goulette F, and Guibas L. KPConv: Flexible and Deformable Convolution for Point Clouds. In: *Proc. of the IEEE/CVF Intl. Conf. on Computer Vision (ICCV)*. 2019.

37. Reynolds DA. Gaussian Mixture Models. In: *Encyclopedia of Biometrics*. 2018.

38. Menon R, Zaenker T, Dengler N, and Bennewitz M. NBV-SC: Next Best View Planning Based on Shape Completion for Fruit Mapping and Reconstruction. In: *Proc. of the IEEE/RSJ Intl. Conf. on Intelligent Robots and Systems (IROS)*. 2023.

39. Hegde D, Valanarasu JMJ, and Patel V. Clip goes 3d: Leveraging prompt tuning for language grounded 3d recognition. In: *Proc. of the IEEE/CVF Conf. on Computer Vision and Pattern Recognition (CVPR)*. 2023.

40. Jayasumana S, Ramalingam S, Veit A, Glasner D, Chakrabarti A, and Kumar S. Rethinking fid: Towards a better evaluation metric for image generation. In: *Proc. of the IEEE/CVF Conf. on Computer Vision and Pattern Recognition (CVPR)*. 2024.

41. Kynkäänniemi T, Karras T, Laine S, Lehtinen J, and Aila T. Improved precision and recall metric for assessing generative models. In: *Proc. of the Conf. on Neural Information Processing Systems (NeurIPS)*. 2019.

42. Marks E, Bömer J, Magistri F, Sah A, Behley J, and Stachniss C. BonnBeetClouds3D: A Dataset Towards Point Cloud-Based Organ-Level Phenotyping of Sugar Beet Plants Under Real Field Conditions. In: *Proc. of the IEEE/RSJ Intl. Conf. on Intelligent Robots and Systems (IROS)*. 2024.

43. Schunck D, Magistri F, Rosu R, et al. Pheno4D: A spatio-temporal dataset of maize and tomato plant point clouds for phenotyping and advanced plant analysis. PLOS ONE 2021;16:1–18.

44. Roggiolani G, Magistri F, Guadagnino T, Behley J, and Stachniss C. Unsupervised Pre-Training for 3D Leaf Instance Segmentation. IEEE Robotics and Automation Letters (RA-L) 11 2023;8.

45. Nunes L, Marcuzzi R, Mersch B, Behley J, and Stachniss C. Scaling Diffusion Models to Real-World 3D LiDAR Scene Completion. In: *Proc. of the IEEE/CVF Conf. on Computer Vision and Pattern Recognition (CVPR)*. 2024.

46. Kingma D and Ba J. Adam: A Method for Stochastic Optimization. In: *Proc. of the Intl. Conf. on Learning Representations (ICLR)*. 2015.

47. Heusel M, Ramsauer H, Unterthiner T, Nessler B, and Hochreiter S. GANs Trained by a Two Time-Scale Update Rule Converge to a Local Nash Equilibrium. In: *Proc. of the Conf. Neural Information Processing Systems (NIPS)*. 2017.

48. Mohammadi SS, Wang Y, and Del Bue A. Pointview-GCN: 3D Shape Classification With Multi-View Point Clouds. In: *Proc. of the IEEE Intl. Conf. on Image Processing (ICIP)*. 2021.

49. Heusel M, Ramsauer H, Unterthiner T, Nessler B, and Hochreiter S. Gans trained by a two time-scale update rule converge to a local nash equilibrium. Proc. of the Conf. on Neural Information Processing Systems (NeurIPS) 2017;30.

50. Sajjadi MS, Bachem O, Lucic M, Bousquet O, and Gelly S. Assessing generative models via precision and recall. Proc. of the Conf. on Neural Information Processing Systems (NeurIPS) 2018;31.

51. Bińkowski M, Sutherland DJ, Arbel M, and Gretton A. Demystifying MMD GANs. In: *Proc. of the Intl. Conf. on Learning Representations (ICLR)*. 2018.

52. Miyato T, Kataoka T, Koyama M, and Yoshida Y. Spectral Normalization for Generative Adversarial Networks. In: *Proc. of the Intl. Conf. on Learning Representations (ICLR)*. 2018.

53. Hart PE, Nilsson NJ, and Raphael B. A formal basis for the heuristic determination of minimum cost paths. IEEE Trans. on Systems Science and Cybernetics 1968;4:100–7.

54. Bernardini F, Mittleman J, Rushmeier H, Silva C, and Taubin G. The ball-pivoting algorithm for surface reconstruction. tvcg 1999;5:349–59.

55. Chang A, Funkhouser T, Guibas L, et al. ShapeNet: An Information-Rich 3D Model Repository. Tech. rep. arXiv:1512.03012 [cs.GR]. Stanford University — Princeton University — Toyota Technological Institute at Chicago, 2015.

56. Deitke M, Schwenk D, Salvador J, Weihs L, Michel O, VanderBilt E, Schmidt L, Ehsani K, Kembhavi A, and Farhadi A. Objaverse: A universe of annotated 3d objects. In: *Proc. of the IEEE/CVF Conf. on Computer Vision and Pattern Recognition (CVPR)*. 2023.
